# Leveraging transdiagnostic genetic liability to psychiatric disorders to dissect clinical outcomes of anorexia nervosa

**DOI:** 10.1038/s41380-025-03264-x

**Published:** 2025-09-23

**Authors:** Zheng-An Lu, Alexander Ploner, Andreas Birgegård, Eating Disorders Working Group of the Psychiatric Genomics Consortium, Andreas Birgegård, Nancy L. Pedersen, Shuyang Yao, Julien Bryois, Virpi M. Leppä, Paul Lichtenstein, Jessica H. Baker, Stephanie Zerwas, Laura M. Thornton, Maria C. La Via, Melissa A. Munn-Chernoff, Bochao Danae Lin, Jurjen Luykx, Roger A. H. Adan, Unna N. Danner, Lars Alfredsson, Tetsuya Ando, Ole A. Andreassen, Morten Mattingsdal, Harald Aschauer, Vladimir Bencko, Andrew W. Bergen, Wade H. Berrettini, Joseph M. Boden, L. John Horwood, Ilka Boehm, Stefan Ehrlich, Christopher S. Franklin, Ioanna Tachmazidou, Vesna Boraska Perica, Steven Crawford, Harry Brandt, Anne Farmer, Peter McGuffin, Gursharan Kalsi, Gerome Breen, Héléna A. Gaspar, Jonathan R. I. Coleman, Marion Roberts, Kirstin L. Purves, Ken B. Hanscombe, Roland Burghardt, Laura Carlberg, Maurizio Clementi, Matteo Cassina, Monica Forzan, Sven Cichon, Stefan Herms, Andreas J. Forstner, Roger D. Cone, Philippe Courtet, Sébastien Guillaume, Scott Crow, Paola Giusti-Rodríquez, Jin P. Szatkiewicz, James J. Crowley, Patrick F. Sullivan, Oliver S. P. Davis, Martina deZwaan, George Dedoussis, Ioanna Ntalla, Angela Favaro, Daniela Degortes, Elena Tenconi, Janiece E. DeSocio, Danielle M. Dick, Dimitris Dikeos, Fragiskos Gonidakis, Christian Dina, Monika Dmitrzak-Weglarz, Elisa Docampo, Geòrgia Escaramís, Monica Gratacos Mayora, Laramie E. Duncan, Philibert Duriez, Philip Gorwood, Nicolas Ramoz, Karin Egberts, Krista Fischer, Tõnu Esko, Thomas Espeseth, Xavier Estivill, Fernando Fernández-Aranda, Susana Jiménez-Murcia, Manfred M. Fichter, James A. B. Floyd, Manuel Föcker, Lenka Foretova, Marie Navratilova, Steven Gallinger, Giovanni Gambaro, Ina Giegling, Dan Rujescu, Scott Gordon, Nicholas G. Martin, Jakob Grove, Yiran Guo, Dong Li, Hakon Hakonarson, Katherine A. Halmi, Lorraine Southam, Konstantinos Hatzikotoulas, Joanna Hauser, Johannes Hebebrand, Triinu Peters, Anke Hinney, Sietske G. Helder, Anjali Henders, Beate Herpertz-Dahlmann, Jochen Seitz, Wolfgang Herzog, Christopher Hübel, Zeynep Yilmaz, Jiayi Xu, Jessica S. Johnson, Laura M. Huckins, Dalila Pinto, James I. Hudson, Hartmut Imgart, Hidetoshi Inoko, Vladimir Janout, Craig Johnson, Jennifer Jordan, Sara Marsal, Antonio Julià, Hana Papezova, Deborah Kaminská, Allan S. Kaplan, James L. Kennedy, Jaakko Kaprio, Elisabeth Widen, Leila Karhunen, Andreas Karwautz, Gudrun Wagner, Martien J. H. Kas, Walter H. Kaye, Martin A. Kennedy, Anna Keski-Rahkonen, Kirsty Kiezebrink, Youl-Ri Kim, Katherine M. Kirk, Sarah E. Medland, Richard Parker, Lars Klareskog, Leonid Padyukov, Kelly L. Klump, Gun Peggy S. Knudsen, Janne T. Larsen, Liselotte V. Petersen, Preben Bo Mortensen, Stephanie Le Hellard, Lisa Lilenfeld, Jolanta Lissowska, Astri J. Lundervold, Pierre J. Magistretti, Alessio Maria Monteleone, Mario Maj, Katrin Mannik, Christian R. Marshall, Manuel Mattheisen, Sara McDevitt, Andres Metspalu, P. Eline Slagboom, Ingrid Meulenbelt, Nadia Micali, James Mitchell, Karen Mitchell, Palmiero Monteleone, Grant W. Montgomery, Benedetta Nacmias, Sandro Sorbi, David C. Whiteman, Catherine M. Olsen, Roel A. Ophoff, Julie O’Toole, Aarno Palotie, Jacques Pantel, John F. Pearson, Anu Raevuori, Ted Reichborn-Kjennerud, Valdo Ricca, Samuli Ripatti, Stephan Ripke, Alessandro Rotondo, Filip Rybakowski, Paolo Santonastaso, André Scherag, Stephen W. Scherer, Ulrike Schmidt, Janet Treasure, Nicholas J. Schork, Alexandra Schosser, Lenka Slachtova, Margarita C. T. Slof-Op’t, Eric F. van Furth, Marta Tyszkiewicz-Nwafor, Agnieszka Slopien, Nicole Soranzo, Vidar W. Steen, Michael Strober, Garret D. Stuber, Beata Świątkowska, Friederike I. Tam, Alfonso Tortorella, Federica Tozzi, Artemis Tsitsika, Konstantinos Tziouvas, Annemarie van Elburg, Tracey D. Wade, Hunna J. Watson, Thomas Werge, H-Erich Wichmann, D. Blake Woodside, Eleftheria Zeggini, Stephan Zipfel, Sarah L. Maguire, Mikael Landén, Cynthia M. Bulik, Mikael Landén, Cynthia M. Bulik, Sarah E. Bergen

**Affiliations:** 1 Department of Medical Epidemiology and Biostatistics, Karolinska Institutet, Stockholm, Sweden; 2 Institute of Neuroscience and Physiology, Sahlgrenska Academy at University of Gothenburg, Gothenburg, Sweden; 3 Department of Psychiatry, University of North Carolina at Chapel Hill, Chapel Hill, NC USA; 4 Department of Nutrition, University of North Carolina at Chapel Hill, Chapel Hill, NC USA; 5 Brain Center Rudolf Magnus, Department of Translational Neuroscience, University Medical Center Utrecht, Utrecht, The Netherlands; 6 Center for Eating Disorders Rintveld, Altrecht Mental Health Institute, Zeist, The Netherlands; 7 Sahlgrenska Academy, University of Gothenburg, Gothenburg, Sweden; 8 Institute of Environmental Medicine, Karolinska Institutet, Stockholm, Sweden; 9 Department of Behavioral Medicine, National Institute of Mental Health, National Center of Neurology and Psychiatry, Kodaira, Tokyo Japan; 10 NORMENT KG Jebsen Centre, Division of Mental Health and Addiction, University of Oslo, Oslo University Hospital, Oslo, Norway; 11 Biopsychosocial Corporation, Vienna, Austria; 12 First Faculty of Medicine, Institute of Hygiene and Epidemiology, Charles University, Prague, Czech Republic; 13 BioRealm, LLC, Walnut, CA USA; 14 Oregon Research Institute, Eugene, OR USA; 15 Department of Psychiatry, Center for Neurobiology and Behavior, University of Pennsylvania Perelman School of Medicine, Philadelphia, PA USA; 16 Christchurch Health and Development Study, University of Otago, Christchurch, New Zealand; 17 Division of Psychological and Social Medicine and Developmental Neurosciences, Faculty of Medicine, Technische Universität Dresden, Dresden, Germany; 18 Wellcome Sanger Institute, Wellcome Genome Campus, Hinxton, CA UK; 19 Department of Medical Biology, School of Medicine, University of Split, Split, Croatia; 20 The Center for Eating Disorders at Sheppard Pratt, Baltimore, MD USA; 21 Social, Genetic and Developmental Psychiatry (SGDP) Centre, King’s College London, London, UK; 22 National Institute for Health Research Biomedical Research Centre, King’s College London and South London and Maudsley National Health Service Trust, London, UK; 23 Klinikum Frankfurt/Oder, Frankfurt, Germany; 24 Medical University of Vienna, Vienna, Austria; 25 Clinical Genetics Unit, Department of Woman and Child Health, University of Padova, Padova, Italy; 26 Institute of Medical Genetics and Pathology, University Hospital Basel, Basel, Switzerland; 27 Department of Biomedicine, University of Basel, Basel, Switzerland; 28 Institute of Neuroscience and Medicine (INM-1), Research Center Juelich, Juelich, Germany; 29 Institute of Human Genetics, University of Bonn, School of Medicine and University Hospital Bonn, Bonn, Germany; 30 Centre for Human Genetics, University of Marburg, Marburg, Germany; 31 Life Sciences Institute and Department of Molecular and Integrative Physiology, University of Michigan, Ann Arbor, MI USA; 32 Department of Emergency Psychiatry and Post-Acute Care, CHRU Montpellier, University of Montpellier, Montpellier, France; 33 Department of Psychiatry, University of Minnesota, Minneapolis, MN USA; 34 Department of Genetics, University of North Carolina at Chapel Hill, Chapel Hill, NC USA; 35 Department of Clinical Neuroscience, Karolinska Institutet, Stockholm, Sweden; 36 MRC Integrative Epidemiology Unit, University of Bristol, Bristol, UK; 37 Bristol Medical School, University of Bristol, Bristol, UK; 38 The Alan Turing Institute, London, UK; 39 Department of Psychosomatic Medicine and Psychotherapy, Hannover Medical School, Hannover, Germany; 40 Department of Nutrition and Dietetics, Harokopio University, Athens, Greece; 41 Department of Neurosciences, University of Padova, Padova, Italy; 42 College of Nursing, Seattle University, Seattle, WA USA; 43 Department of Psychology, Virginia Commonwealth University, Richmond, VA USA; 44 College Behavioral and Emotional Health Institute, Virginia Commonwealth University, Richmond, VA USA; 45 Department of Human and Molecular Genetics, Virginia Commonwealth University, Richmond, VA USA; 46 First Department of Psychiatry, National and Kapodistrian University of Athens, Medical School, Eginition Hospital, Athens, Greece; 47 L’institut du thorax, INSERM, CNRS, UNIV Nantes, Nantes, France; 48 Department of Psychiatric Genetics, Poznan University of Medical Sciences, Poznan, Poland; 49 Barcelona Institute of Science and Technology, Barcelona, Spain; 50 Universitat Pompeu Fabra, Barcelona, Spain; 51 Centro de Investigación Biomédica en Red en Epidemiología y Salud Pública (CIBERESP), Barcelona, Spain; 52 Department of Psychiatry and Behavioral Sciences, Stanford University, Stanford, CA USA; 53 GHU Paris Psychiatrie et Neurosciences, CMME, Paris Descartes University, Paris, France; 54 Univ. Paris Cité, Institut de psychiatrie et Neurosciences de Paris, INSERM, U1266, Vulnerability of psychiatric and addictive disorders, Paris, France; 55 Department of Child and Adolescent Psychiatry, Psychosomatics and Psychotherapy, University Hospital of Würzburg, Centre for Mental Health, Würzburg, Germany; 56 Estonian Genome Center, University of Tartu, Tartu, Estonia; 57 Program in Medical and Population Genetics, Broad Institute of Massachusetts Institute of Technology and Harvard University, Cambridge, MA USA; 58 Department of Psychology, University of Oslo, Oslo, Norway; 59 Bjørknes College, Oslo, Norway; 60 Genomics and Disease, Bioinformatics and Genomics Programme, Centre for Genomic Regulation, Barcelona, Spain; 61 Department of Psychiatry, University Hospital of Bellvitge – IDIBELL and CIBERobn, Barcelona, Spain; 62 Department of Clinical Sciences, School of Medicine, University of Barcelona, Barcelona, Spain; 63 Department of Psychiatry and Psychotherapy, Ludwig-Maximilians-University (LMU), Munich, Germany; 64 Schön Klinik Roseneck affiliated with the Medical Faculty of the University of Munich, Munich, Germany; 65 Genomics plc, Genomics PLC, Oxford, UK; 66 Department of Child and Adolescent Psychiatry, University of Münster, Münster, Germany; 67 Department of Cancer, Epidemiology and Genetics, Masaryk Memorial Cancer Institute, Brno, Czech Republic; 68 Department of Surgery, Faculty of Medicine, University of Toronto, Toronto, ON Canada; 69 Division of Nephrology and Dialysis, Department of Medicine, AOVR, Ospedale Maggiore, Verona, Italy; 70 Department of Psychiatry, Psychotherapy and Psychosomatics, Martin Luther University of Halle-Wittenberg, Halle (Saale), Germany; 71 QIMR Berghofer Medical Research Institute, Brisbane, QLD Australia; 72 Department of Biomedicine, Aarhus University, Aarhus, Denmark; 73 The Lundbeck Foundation Initiative for Integrative Psychiatric Research (iPSYCH), Aarhus, Denmark; 74 Centre for Integrative Sequencing, iSEQ, Aarhus University, Aarhus, Denmark; 75 Bioinformatics Research Centre, Aarhus University, Aarhus, Denmark; 76 Center for Applied Genomics, Children’s Hospital of Philadelphia, Philadelphia, PA USA; 77 Department of Pediatrics, University of Pennsylvania Perelman School of Medicine, Philadelphia, PA USA; 78 Department of Psychiatry, Weill Cornell Medical College, New York, NY USA; 79 Institute of Translational Genomics, Helmholtz Zentrum München – German Research Centre for Environmental Health, Neuherberg, Germany; 80 Department of Adult Psychiatry, Poznan University of Medical Sciences, Poznan, Poland; 81 Department of Child and Adolescent Psychiatry, University Hospital Essen, University of Duisburg-Essen, Essen, Germany; 82 Zorg op Orde, Delft, The Netherlands; 83 Institute for Molecular Bioscience, University of Queensland, Brisbane, QLD Australia; 84 Department of Child and Adolescent Psychiatry, Psychosomatics and Psychotherapy, RWTH Aachen University, Aachen, Germany; 85 Department of General Internal Medicine and Psychosomatics, Heidelberg University Hospital, Heidelberg University, Heidelberg, Germany; 86 National Centre for Register-Based Research, Aarhus BSS, Aarhus University, Aarhus, Denmark; 87 Department of Psychiatry, Yale School of Medicine, New Haven, CT 06510 USA; 88 Department of Psychiatry, and Genetics and Genomic Sciences, Icahn School of Medicine at Mount Sinai, New York, NY USA; 89 Seaver Autism Center for Research and Treatment, Icahn School of Medicine at Mount Sinai, New York, NY USA; 90 Mental Illness Research, Education and Clinical Centers, James J. Peters Department of Veterans Affairs Medical Center, Bronx, NY USA; 91 Biological Psychiatry Laboratory, McLean Hospital/Harvard Medical School, Boston, MA USA; 92 Eating Disorders Unit, Parklandklinik, Bad Wildungen, Germany; 93 Department of Molecular Life Science, Division of Basic Medical Science and Molecular Medicine, School of Medicine, Tokai University, Isehara, Japan; 94 Faculty of Health Sciences, Palacky University, Olomouc, Czech Republic; 95 Eating Recovery Center, Denver, CO USA; 96 Department of Psychological Medicine, University of Otago, Christchurch, New Zealand; 97 Canterbury District Health Board, Christchurch, New Zealand; 98 Rheumatology Research Group, Vall d’Hebron Research Institute, Barcelona, Spain; 99 First Faculty of Medicine, Department of Psychiatry, Charles University, Prague, Czech Republic; 100 Centre for Addiction and Mental Health, Toronto, ON Canada; 101 Institute of Medical Science, University of Toronto, Toronto, ON Canada; 102 Department of Psychiatry, University of Toronto, Toronto, ON Canada; 103 Institute for Molecular Medicine Finland FIMM, HiLIFE, Helsinki Institute of Life Science, University of Helsinki, Helsinki, Finland; 104 Institute of Public Health and Clinical Nutrition, Department of Clinical Nutrition, University of Eastern Finland, Kuopio, Finland; 105 Eating Disorders Unit, Department of Child and Adolescent Psychiatry, Medical University of Vienna, Vienna, Austria; 106 Groningen Institute for Evolutionary Life Sciences, University of Groningen, Groningen, The Netherlands; 107 Department of Psychiatry, University of California San Diego, San Diego, California, USA; 108 Department of Pathology and Biomedical Science, University of Otago, Christchurch, New Zealand; 109 Department of Public Health, University of Helsinki, Helsinki, Finland; 110 Institute of Applied Health Sciences, School of Medicine, Medical Sciences and Nutrition, University of Aberdeen, Aberdeen, UK; 111 Department of Psychiatry, Seoul Paik Hospital, Inje University, Seoul, Korea; 112 Division of Rheumatology, Department of Medicine, Center for Molecular Medicine, Karolinska Institutet and Karolinska University Hospital, Stockholm, Sweden; 113 Department of Psychology, Michigan State University, East Lansing, MI USA; 114 Department of Mental Disorders, Norwegian Institute of Public Health, Oslo, Norway; 115 Centre for Integrated Register-based Research (CIRRAU), Aarhus University, Aarhus, Denmark; 116 Dr. Einar Martens Research Group for Biological Psychiatry, Center for Medical Genetics and Molecular Medicine, Haukeland University Hospital, Bergen, Norway; 117 Department of Clinical Medicine, Laboratory Building, Haukeland University Hospital, Bergen, Norway; 118 The Chicago School of Professional Psychology, Washington D.C., USA; 119 Department of Cancer Epidemiology and Prevention, M. Sklodowska-Curie National Research Institute of Oncology, Warsaw, Poland; 120 Department of Biological and Medical Psychology, University of Bergen, Bergen, Norway; 121 BESE Division, KAUST, KSA, King Abdullah University of Science and Technology, Thuwal, Saudi Arabia; 122 Department of Psychiatry, University of Lausanne-University Hospital of Lausanne (UNIL-CHUV), Lausanne, Switzerland; 123 Department of Psychiatry, University of Campania “Luigi Vanvitelli”, Naples, Italy; 124 Center for Integrative Genomics, University of Lausanne, Lausanne, Switzerland; 125 Department of Paediatric Laboratory Medicine, Division of Genome Diagnostics, The Hospital for Sick Children, Toronto, ON Canada; 126 Center for Psychiatry Research, Stockholm Health Care Services, Stockholm City Council, Stockholm, Sweden; 127 Department of Psychiatry, Psychosomatics and Psychotherapy, University of Würzburg, Würzburg, Germany; 128 Department of Psychiatry, University College Cork, Cork, Ireland; 129 Child and Adolescent Regional Eating Disorder Service (CAREDS), Health Service Executive South, Cork, Ireland; 130 Institute of Molecular and Cell Biology, University of Tartu, Tartu, Estonia; 131 Molecular Epidemiology Section, Department of Biomedical Datasciences, Leiden University Medical Centre, Leiden, The Netherlands; 132 Institute of Biological Psychiatry, Mental Health Center Sct. Hans, Copenhagen University Hospital, Mental Health Services Copenhagen, Roskilde, Denmark; 133 Center for Eating and feeding Disorders Research, Mental Health Center Ballerup, Copenhagen University Hospital, Mental Health Services Copenhagen, Copenhagen, Denmark; 134 Department of Psychiatry and Behavioral Science, University of North Dakota School of Medicine and Health Sciences, Fargo, ND USA; 135 National Center for PTSD, VA Boston Healthcare System, Boston, MA USA; 136 Department of Psychiatry, Boston University School of Medicine, Boston, MA USA; 137 Department of Medicine, Surgery and Dentistry “Scuola Medica Salernitana”, University of Salerno, Salerno, Italy; 138 Queensland Brain Institute, University of Queensland, Brisbane, QLD Australia; 139 Department of Neuroscience, Psychology, Drug Research and Child Health (NEUROFARBA), University of Florence, Florence, Italy; 140 IRCCS Fondazione Don Carlo Gnocchi, Florence, Italy; 141 Population Health Department, QIMR Berghofer Medical Research Institute, Brisbane, QLD Australia; 142 Center for Neurobehavioral Genetics, Semel Institute for Neuroscience and Human Behavior, University of California Los Angeles, Los Angeles, CA USA; 143 Department of Psychiatry, Erasmus MC, University Medical Center Rotterdam, Rotterdam, The Netherlands; 144 Kartini Clinic, Portland, OR USA; 145 Center for Human Genome Research, Massachusetts General Hospital, Boston, MA USA; 146 Centre of Psychiatry and Neuroscience, INSERM U1124, Université de Paris, Paris, France; 147 Biostatistics and Computational Biology Unit, University of Otago, Christchurch, New Zealand; 148 Department of Adolescent Psychiatry, Helsinki University Hospital, Helsinki, Finland; 149 Institute of Clinical Medicine, University of Oslo, Oslo, Norway; 150 Department of Health Science, University of Florence, Florence, Italy; 151 Department of Biometry, University of Helsinki, Helsinki, Finland; 152 Analytic and Translational Genetics Unit, Department of Medicine, Massachusetts General Hospital and Harvard Medical School, Boston, MA USA; 153 Stanley Center for Psychiatric Research, Broad Institute of the Massachusetts Institute of Technology and Harvard University, Cambridge, MA USA; 154 Department of Psychiatry and Psychotherapy, Charité – Universitätsmedizin, Berlin, Germany; 155 Department of Psychiatry, Neurobiology, Pharmacology, and Biotechnologies, University of Pisa, Pisa, Italy; 156 Department of Psychiatry, Poznan University of Medical Sciences, Poznan, Poland; 157 Department of Neurosciences, Padua Neuroscience Center, University of Padova, Padova, Italy; 158 Institute of Medical Statistics, Computer and Data Sciences, Jena University Hospital, Jena, Germany; 159 Department of Genetics and Genomic Biology, The Hospital for Sick Children, Toronto, ON Canada; 160 McLaughlin Centre, University of Toronto, Toronto, ON Canada; 161 Institute of Psychiatry, Psychology and Neuroscience, Psychological Medicine, King’s College London, London, UK; 162 J. Craig Venter Institute (JCVI), La Jolla, CA USA; 163 Department of Psychiatry and Psychotherapy, Medical University of Vienna, Vienna, Austria; 164 First Faculty of Medicine, Department of Biology and Medical Genetics, Charles University, Prague, Czech Republic; 165 Center for Eating Disorders Ursula, Rivierduinen, Leiden, The Netherlands; 166 Department of Psychiatry, Leiden University Medical Centre, Leiden, The Netherlands; 167 Department of Child and Adolescent Psychiatry, Poznan University of Medical Sciences, Poznan, Poland; 168 Donor Health and Genomics, National Institute for Health Research Blood and Transplant Unit, Cambridge, UK; 169 Division of Cardiovascular Medicine, British Heart Foundation Centre of Excellence, Cambridge, UK; 170 Department of Haematology, University of Cambridge, Cambridge, UK; 171 Center for Medical Genetics and Molecular Medicine, Haukeland University Hospital, Bergen, Norway; 172 Department of Clinical Science, University of Bergen, Bergen, Norway; 173 Department of Psychiatry and Biobehavioral Science, Semel Institute for Neuroscience and Human Behavior, University of California Los Angeles, Los Angeles, CA USA; 174 David Geffen School of Medicine, University of California Los Angeles, Los Angeles, CA USA; 175 Department of Cell Biology and Physiology, University of North Carolina at Chapel Hill, Chapel Hill, NC USA; 176 Department of Environmental Epidemiology, Nofer Institute of Occupational Medicine, Lodz, Poland; 177 Eating Disorders Research and Treatment Center, Department of Child and Adolescent Psychiatry, Faculty of Medicine, Technische Universität Dresden, Dresden, Germany; 178 Department of Psychiatry, University of Perugia, Perugia, Italy; 179 Brain Sciences Department, Stremble Ventures, Limassol, Cyprus; 180 Adolescent Health Unit, Second Department of Pediatrics, “P. & A. Kyriakou” Children’s Hospital, University of Athens, Athens, Greece; 181 Pediatric Intensive Care Unit, “P. & A. Kyriakou” Children’s Hospital, University of Athens, Athens, Greece; 182 Faculty of Social and Behavioral Sciences, Utrecht University, Utrecht, The Netherlands; 183 School of Psychology, Flinders University, Adelaide, SA Australia; 184 School of Psychology, Curtin University, Perth, WA Australia; 185 School of Paediatrics and Child Health, University of Western Australia, Perth, WA Australia; 186 Department of Clinical Medicine, University of Copenhagen, Copenhagen, Denmark; 187 Helmholtz Centre Munich – German Research Center for Environmental Health, Munich, Germany; 188 Centre for Mental Health, University Health Network, Toronto, ON Canada; 189 Program for Eating Disorders, University Health Network, Toronto, ON Canada; 190 Technical University of Munich (TUM) and Klinikum Rechts der Isar, TUM School of Medicine, Munich, Germany; 191 Department of Internal Medicine VI, Psychosomatic Medicine and Psychotherapy, University Medical Hospital Tuebingen, Tuebingen, Germany; 192 Centre of Excellence for Eating Disorders (KOMET), University Tuebingen, Tuebingen, Germany; 193 School of Medicine, InsideOut Institute, The University of Sydney, Sydney, NSW Australia

**Keywords:** Genetics, Psychiatric disorders

## Abstract

Anorexia nervosa (AN) has extensive genetic correlations with other psychiatric disorders, and genetic risk for different psychiatric disorders was associated with distinct clinical courses in AN. Uncovering associations between transdiagnostic psychiatric genetic liability and AN outcomes can facilitate its personalized treatment. In this study, we investigated the associations between transdiagnostic psychiatric genetic liability and outcomes of AN. Genomic structural equation models were fitted to genome-wide association data for AN and psychiatric disorders with high genetic correlations with AN (obsessive-compulsive symptoms [OCS], major depressive disorder [MDD], schizophrenia, and anxiety disorders) to extract one shared and five trait-specific genetic components. Next, we calculated the polygenic risk scores (PRS) for these components, including PRS_shared_, PRS_AN-specific_, PRS_OCS-specific_, PRS_MDD-specific_, PRS_SCZ-specific_ and PRS_ANX-specific_, which index the shared genetic liability to all five psychiatric traits, and genetic liability specific to AN, OCS, MDD, SCZ and ANX, respectively. We then tested associations between these PRSs and clinical outcomes reported between 1997 and 2018 among AN cases from the Anorexia Nervosa Genetics Initiative (ANGI), linked to Swedish National Registers. The clinical outcomes included cumulative disease burden (i.e., number of diagnoses, medication prescriptions, and inpatient days), risks of psychiatric comorbidities, and AN symptomatology. Among 4028 included AN cases, the mean (SD) birth year was 1985 (9), and 3947 (98.0%) were female. Within AN, +1 SD increase of PRS_shared_ was associated with 9–39% excess risk of disease burden and psychiatric comorbidity, whereas the associations between PRS_AN-specific_ and most clinical outcomes were statistically non-significant. +1 SD increase of PRS_MDD-specific_ was associated with 3–29% increased risk of AN disease burden. Our findings show that shared psychiatric liability is associated with more adverse AN outcomes, whereas AN-specific liability is not a good indicator for its clinical course. This study provides a novel perspective on factors influencing heterogeneity in AN clinical course.

## Introduction

Anorexia nervosa (AN) is a serious and often chronic psychiatric disorder with significant morbidity and mortality [1]. AN is characterized by very low body weight and an intense fear of gaining weight, with a lifetime prevalence of approximately 1% and a full recovery rate of less than 50% [2, 3]. It is a moderately heritable disorder with a twin-based heritability of 50–70% [4]. The latest genome-wide association study (GWAS) identified 8 genomic loci for AN and showed that 17% of variance in liability to AN is attributable to common genetic variants [5].

AN is highly comorbid with other psychiatric disorders [1]. It commonly co-occurs with obsessive-compulsive disorder (OCD) (14%), major depressive disorder (MDD) (73%) and anxiety disorders (ANX) (75%) and is associated with 6-fold greater risk of having schizophrenia (SCZ) [6–9]. Moreover, some clinical features of AN, such as perfectionism and obsessive exercise, have obsessive and compulsive characteristics [8]. Similarly, distorted perceptions of body shape in AN resemble delusional thinking in SCZ [10].

Phenotypic co-aggregation between AN and other psychiatric disorders can be partially explained by genetic factors, as indicated by their extensive genetic overlap. Notable genetic correlations (*r*_g_) based on common variants have been estimated at AN vs. OCD = 0.45, AN vs. MDD = 0.28, AN vs. SCZ = 0.25, and AN vs. ANX = 0.25 [5]. This strong but incomplete overlap indicates the existence of pleiotropic variants influencing multiple disorders as well as variants specific to each disorder.

The clinical course of AN is heterogeneous, with some cases being relatively brief but nearly a third giving way to a severe and enduring profile [1]. Interestingly, we have previously shown that the variance in AN course can be partially explained by genetic liability to other psychiatric disorders: genetic liability to SCZ was associated with distinct phenotypes in AN [11]. Furthermore, the transdiagnostic polygenic risk score (PRS) for AN and OCD demonstrated good performance in predicting the risk of AN [12].

Genomic structural equation modelling (gSEM) is a method to capture the multivariate genetic architecture of genetically correlated traits based on GWAS summary statistics. gSEM is capable of isolating a pleiotropic component representing broad genetic liability in addition to components representing liability specific to each trait. gSEM has been employed to distinguish shared and disorder-specific liability between BIP, MDD, and SCZ; autism spectrum disorder (ASD) and attention-deficit hyperactivity disorder (ADHD); as well as MDD and ANX [13–16]. Individual single nucleotide polymorphisms (SNPs) can be integrated into the gSEM model so that effects of a specific SNP on the shared liability component and trait-specific components can be estimated, allowing the construction of more predictive and valid PRSs [16]. These gSEM-derived PRSs indexing shared and non-shared genetic liabilities to psychiatric disorders can be further utilized to characterize the clinical heterogeneity of a specific disorder [13].

Given the consistently demonstrated symptomatic and genetic overlap between AN and other psychiatric disorders, we used gSEM to extract the shared and specific genetic components of AN and four psychiatric traits with high genetic correlations with AN (obsessive compulsive symptoms [OCS], MDD, SCZ and ANX). We calculated PRSs for these components and tested the associations between these PRSs and AN risk as well as a wide range of clinical outcomes to dissect the heterogeneous outcomes of AN.

## Methods

**Fig. 1:**
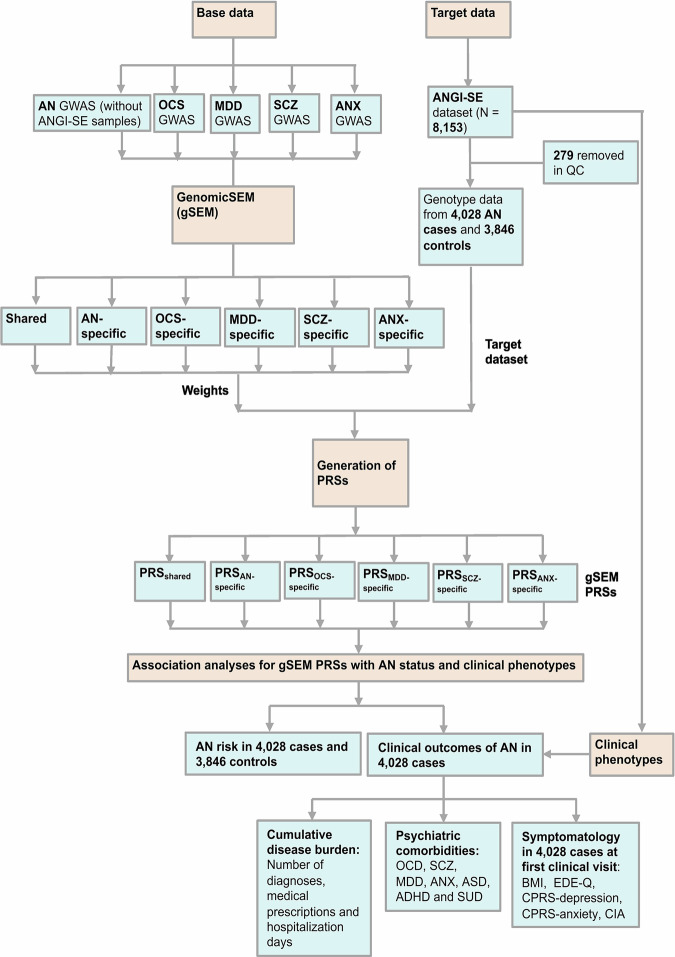
Flow chart of the current study. AN anorexia nervosa, SCZ schizophrenia, OCS obsessive-compulsive symptoms, OCD obsessive-compulsive disorder, MDD major depressive disorder, ANX anxiety disorders, gSEM genomic structural equation model, PRS polygenic risk scores, ASD autism spectrum disorder, ADHD attention-deficit hyperactivity disorder, SUD substance use disorder, BMI body mass index, CIA Clinical Impairment Assessment, CPRS Comprehensive Psychopathological Rating Scale, EDE-Q Eating Disorder Examination Questionnaire.

The work flow of the current study is presented in Fig. 1.

### Source GWAS

The source GWAS summary statistics for AN (without ANGI-SE samples, N_cases_ = 4105, N_controls_ = 3793), OCS and SCZ were acquired from the Psychiatric Genomics Consortium (PGC), whereas the GWAS for MDD was derived from a meta-analysis of six European datasets, and the GWAS for ANX was based on the European subset of a multi-ancestry meta-analysis [5, 17–20]. All individuals in the component data sets were of European ancestry. Detailed information on source GWAS studies is available in Table S1.

### Target genotype data

The target genotype data were acquired from the Swedish arm of the Anorexia Nervosa Genetics Initiative (ANGI-SE). ANGI is an international collaboration committed to collecting genotype and phenotype data from individuals with AN and controls to identify genetic and environmental risk factors of AN [21]. AN status was determined by a DSM-IV-based AN diagnosis or responses to the ED100K-V1 questionnaire [21]. Details regarding recruitment of participants in ANGI-SE are described in the Supplementary Methods.

In the current study, we included all ANGI-SE individuals with available linkage to Swedish National Registers [21]. The quality control (QC) and imputation of the genotype data were performed according to the RICOPILI pipeline, leaving a total of 4028 cases and 3846 controls [22]. Details regarding genotyping and QC are provided in the Supplementary Methods.

### Clinical phenotypes

The data on clinical phenotypes in AN cases were derived from records in Swedish National registers from January 1, 1997 to December 31, 2018, using unique personal identification numbers to link data across registers [23]. We focused on five categories of phenotypes in the current study: (1) clinical diagnoses: general [somatic + psychiatric], somatic and psychiatric; (2) medication prescriptions: general, antipsychotics and antidepressants; (3) inpatient days: any and due to EDs ; (4) psychiatric comorbidities: OCD, SCZ, MDD, ANX, ASD, ADHD, and substance use disorder (SUD); (5) symptomatology of AN at first visit: BMI, Eating Disorder Examination Questionnaire (EDE-Q) score [24], depression subscale score from the Comprehensive Psychopathological Rating Scale (CPRS) [25], anxiety subscale score from the CPRS [25], and Clinical Impairment Assessment (CIA) questionnaire score [26, 27]. Number of clinical diagnoses, medication prescriptions, and inpatient days were defined as cumulative disease burden in the current study.

Data on diagnoses, inpatient days, medication prescriptions, and psychiatric comorbidities were available for all included AN cases, whereas information on AN symptomatology at first visit was only available for 1934 AN cases. Details about the Swedish National Registers that these phenotypes were derived from are described in the Supplementary Methods and Tables S2–S4.

### Statistical analyses

#### GenomicSEM (gSEM)

We used gSEM to construct a common-factor model based on source GWAS datasets and extracted the loading of each SNP on the common factor. We refer to this as the “shared” component, indicating contribution of the SNP to general liability to the five disorders (Fig. S1A and Supplementary Methods). Next, we constructed five models to extract the loading of each SNP on the residual variance from each source GWAS after accounting for the common factor to represent contribution of the SNP to the liability specific to each disorder (Fig. S1B–F and Supplementary Methods). We refer to these as AN-specific, OCS-specific, SCZ-specific, MDD-specific, and ANX-specific effects. gSEM was performed with the R package “GenomicSEM” (“0.0.5”). Code and technical details are available online (https://github.com/GenomicSEM/GenomicSEM/) and in the Supplementary Methods.

#### Generation of PRSs

We generated PRSs using PRSice software (version 2.3.5) [28]. The target dataset was the genotype data from individuals in ANGI-SE. The base summary statistic datasets were the shared and trait-specific effects from gSEM. We clumped the SNPs at r^2^ < 0.1 within 250 kb and aggregated their effects at different p-value thresholds (5e-8, 1e-6, 1e-4, 0.001,0.01, 0.05, 0.1, 0.2, 0.5, 1) using the PRS-PCA method [29]. The final gSEM-derived PRSs utilized in association analyses were the standardized first components derived from principal component analyses (PCA) of PRSs at all thresholds. We performed PCA and standardization with R functions “princomp()” and “scale()”, respectively. Details for PRSs calculation are presented in Supplementary Methods.

#### Association analyses for gSEM PRSs with AN status and clinical phenotypes

To investigate the association between gSEM-derived PRSs and AN status, we conducted logistic regression analyses with binary AN status as the outcome in the 4028 AN cases and 3846 controls. Each gSEM-derived PRS was tested as an exposure variable separately. Odds ratios (ORs) represented the risk estimates +1 standard deviation (SD) increase of PRS.

To investigate the impact of gSEM-derived PRSs on the cumulative disease burden of AN cases, we conducted quasi-Poisson regression analyses with number of unique diagnoses (general, somatic, and psychiatric), number of unique medication prescriptions (any, antipsychotics, and antidepressants), and number of inpatient days (any and due to eating disorders) recorded from January 1, 1997 to December 31, 2018 as outcomes among 4028 AN cases. Each gSEM-derived PRS was tested as an exposure variable for association with these eight outcomes separately. We included a log-time offset term in each model to adjust for differences in follow-up time between individuals. Incidence rate ratios (IRRs) represented the risk estimates +1 SD increase of gSEM-derived PRS.

To investigate the impact of gSEM-derived PRSs on risks of psychiatric comorbidities in AN cases, we conducted Cox regression (survival) analyses with age as the underlying timescale and onset of OCD, SCZ, MDD, ANX, ASD, ADHD, and SUD as outcomes among 4028 AN cases. Each gSEM-derived PRS was tested as an exposure variable for association with these seven outcomes separately. Individuals were followed from January 1, 1997 until onset of disorder, death or December 31, 2018, whichever came first. Hazard ratios (HRs) represented the risk estimates +1 SD increase of gSEM-derived PRS.

To investigate the impact of gSEM-derived PRSs on the symptomatology of AN at first visit in AN cases, we performed linear regression analyses with BMI, EDE-Q scores, CIA scores, CPRS-depression and CPRS-anxiety scores as outcomes among 4028 AN cases. Each gSEM-derived PRS was tested as an exposure variable for association with these five outcomes separately. Regression coefficients represented change in symptoms +1 SD increase of gSEM-derived PRS.

All association analyses were performed in R version 4.2.3. In all regression models, we adjusted for birth year, sex, and the first 10 ancestry-informative principal components. The significance level in all association analyses above was set at two-sided *P* < 0.05. To correct for multiple tests, we also employed a Bonferroni-corrected significance level of two-sided *P* < 0.05/6 = 8.33 × 10^−3^ for association tests with AN status and two-sided *P* < 0.05/120 = 4.17 × 10^−4^ for association tests with clinical outcomes of AN. Details regarding the regression models are presented in the Supplementary Methods and Table S5.

## Results

### Descriptive characteristics of the study population

Of the 4028 AN cases in ANGI-SE, the mean (SD) birth year was 1985 (9), and 3947 (98.0%) were female, whereas among the 3846 controls, the mean (SD) birth year was 1978 (10), and 3776 (98.2%) were female. The descriptive characteristics of the 4028 individuals with AN are presented in Table S6.

### Association between gSEM-derived PRSs and AN status

Only PRS_AN-specific_ and PRS_shared_ were associated with statistically significant increased risk of AN, whereas other gSEM-derived PRSs showed either statistically non-significant or decreased risk of AN after Bonferroni correction (Table 1). In contrast, all unmodified source GWAS PRSs were associated with an elevated risk of AN (Table S7).

**Table 1:** Association between gSEM-derived polygenic risk scores (PRS) and odds of anorexia nervosa (AN) from logistic regression analyses.

PRS	Odds ratio (95% confidence interval)	*P* value
PRS_shared_	1.33 (1.27–1.40)	6.21 × 10^−28^ *
PRS_AN-specific_	1.33 (1.26–1.40)	3.32 × 10^−28^ *
PRS_OCS-specific_	1.03 (0.98–1.08)	1.97 × 10^−1^
PRS_SCZ-specific_	1.06 (1.01–1.11)	2.41 × 10^−2 #^
PRS_MDD-specific_	0.99 (0.95–1.04)	8.04 × 10^−1^
PRS_ANX-specific_	0.88 (0.84–0.92)	3.85 × 10^−7^*

Results were derived from logistic regression models with AN status as the outcome variable. We constructed six models with PRS_shared_, PRS_AN-specific_, PRS_OCS-specific_, PRS_MDD-specific_, PRS_SCZ-specific_ and PRS_ANX-specific_ as the exposure variable, respectively. The analyses were based on 7874 individuals from ANGI-SE (4028 cases and 3846 controls) adjusting for birth year, sex and first 10 ancestry-informative principal components. Odds ratios represent the risk estimates per one standard deviation increase of gSEM-derived PRS. The Bonferroni-corrected significance level was set at *P* < 0.05/6 = 8.33 × 10^−3^.

“*” represents association that remained significant after Bonferroni correction.

“#” represents trending association at *P* < 0.05 but was not significant after Bonferroni correction.

*gSEM* genomic structural equation model, *AN* anorexia nervosa, *SCZ* schizophrenia, *OCS* obsessive-compulsive symptoms, *MDD* major depressive disorder, *ANX* anxiety disorders.

### Association between gSEM-derived PRSs and cumulative disease burden

**Fig. 2:**
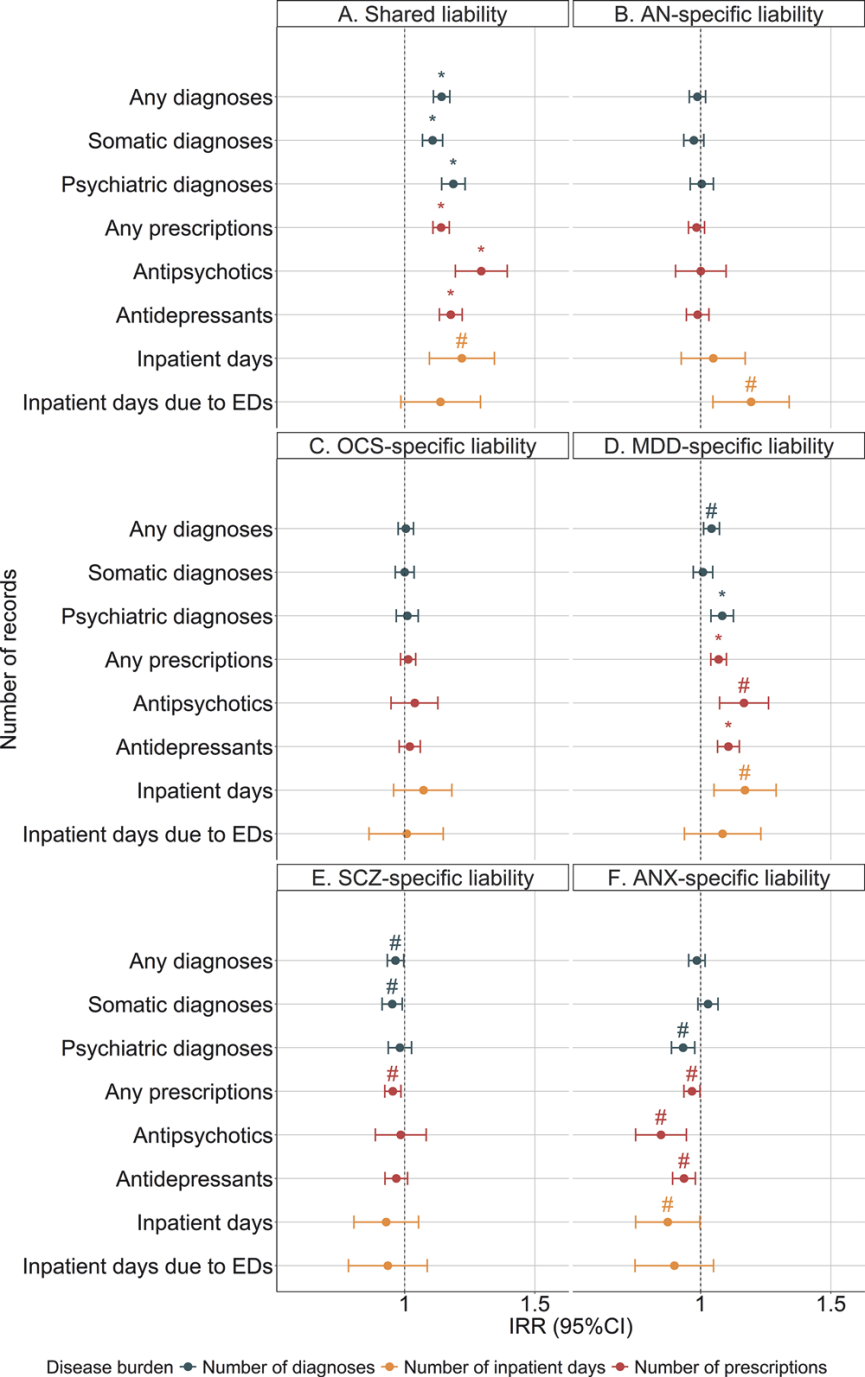
Association between transdiagnostic genetic liabilities and cumulative disease burden from the quasi-Possion models. Results are derived from quasi-Possion regression models based on 4028 AN cases with number of unique clinical diagnoses (any, psychiatric, somatic), prescriptions (any, antipsychotics, antidepressants) and inpatient days (any, due to EDs) as outcomes. For each of the eight outcomes, we constructed six models with PRS_shared_ (**A**), PRS_AN-specific_ (**B**), PRS_OCS-specific_ (**C**), PRS_MDD-specific_ (**D**), PRS_SCZ-specific_ (**E**) and PRS_ANX-specific_ (**F**) as exposure variable, respectively. Incidence rate ratios indicate the risk estimates for +1 SD increase of PRS. Sex, birth year and first 10 ancestry-informative principal components were adjusted for in all models. The points represent incidence rate ratio estimates, and the error bars indicate 95% confidence intervals. Blue points represent effect estimates for clinical diagnoses, red points represent effects for medication prescriptions and yellow points represent effects for inpatient days. “*” represents association that remained significant after Bonferroni correction. “#” represents trending association at *P* < 0.05 but was not significant after Bonferroni correction. PRS polygenic risk scores, EDs eating disorders, IRR incidence rate ratio, 95%CI 95% confidence interval, AN anorexia nervosa, SCZ schizophrenia, OCS obsessive-compulsive symptoms, MDD major depressive disorder, ANX anxiety disorders.

PRS_shared_ was associated with a higher risk for cumulative disease burden (Fig. 2A; Table S8): +1 SD increase in PRS_shared_ was associated with receiving 12% (IRR, 1.12 95%CI, 1.09–1.15; *P* = 1.82 × 10^−18^) more general diagnoses, 16% (IRR, 1.16; 95%CI, 1.12–1.21; *P* = 5.46 × 10^−16^) more psychiatric diagnoses and 9% more somatic diagnoses (IRR, 1.09; 95%CI, 1.06–1.13; *P* = 7.01 × 10^−8^) at *P* < 0.05. For medication prescriptions, +1 SD increase in PRS_shared_ was associated with 12% (IRR, 1.12; 95%CI, 1.09–1.15; *P* = 1.80 × 10^−18^) more general prescriptions, 27% (IRR, 1.27; 95%CI. 1.17–1.38; *P* = 8.06 × 10^−9^) more antipsychotic prescriptions, and 15% (IRR, 1.15; 95%CI, 1.11–1.20; *P* = 3.78 × 10^−15^) more antidepressant prescriptions at *P* < 0.05. PRS_shared_ was also associated with having more inpatient days due to any illness (IRR, 1.19; 95%CI, 1.08–1.32; *P* = 5.79 × 10^−4^) at *P* < 0.05. After Bonferroni correction, all these associations remained significant except for the association with inpatient days.

PRS_AN-specific_ was not statistically significantly associated with most cumulative disease burden outcomes at *P* < 0.05 (Fig. 2B; Table S8), except for a 17% increased risk of inpatient days due to EDs (IRR, 1.17; 95%CI, 1.04–1.32; *P* = 9.75 × 10^−3^). However, it became non-significant after Bonferroni correction. No statistically significant association was detected between PRS_OCS-specific_ and cumulative disease burden at *P* < 0.05 (Fig. 2C; Table S8).

PRS_MDD-specific_ was associated with generally elevated risk for cumulative disease burden (Fig. 2D; Table S8): +1 SD increase in PRS_MDD-specific_ was associated with receiving 3% more general diagnoses (IRR, 1.03; 95%CI, 1.01–1.06; *P* = 7.58 × 10^−3^) and 7% more psychiatric diagnoses (IRR, 1.07; 95%CI, 1.03–1.11; *P* = 1.87 × 10^−4^), 6% more general prescriptions (IRR, 1.06; 95%CI, 1.03–1.08; *P* = 6.85 × 10^−6^), 14% more antipsychotic prescriptions (IRR, 1.14; 95%CI, 1.06–1.24; *P* = 5.43 × 10^−4^) and 9% more antidepressant prescriptions (IRR, 1.09; 95%CI, 1.05–1.13; *P* = 6.63 × 10^−7^) as well as 15% more inpatient days (IRR, 1.15; 95%CI, 1.04–1.26; *P* = 5.31 × 10^−3^) at *P* < 0.05. Only the associations with psychiatric diagnoses, any prescriptions and antidepressant prescriptions survived the Bonferroni correction.

PRS_SCZ-specific_ and PRS_ANX-specific_ were generally associated with a lower risk for cumulative disease burden, but neither was statistically significant after Bonferroni correction (Fig. 2E, F; Table S8).

### Association between gSEM-derived PRSs and risk of psychiatric comorbidities

**Fig. 3:**
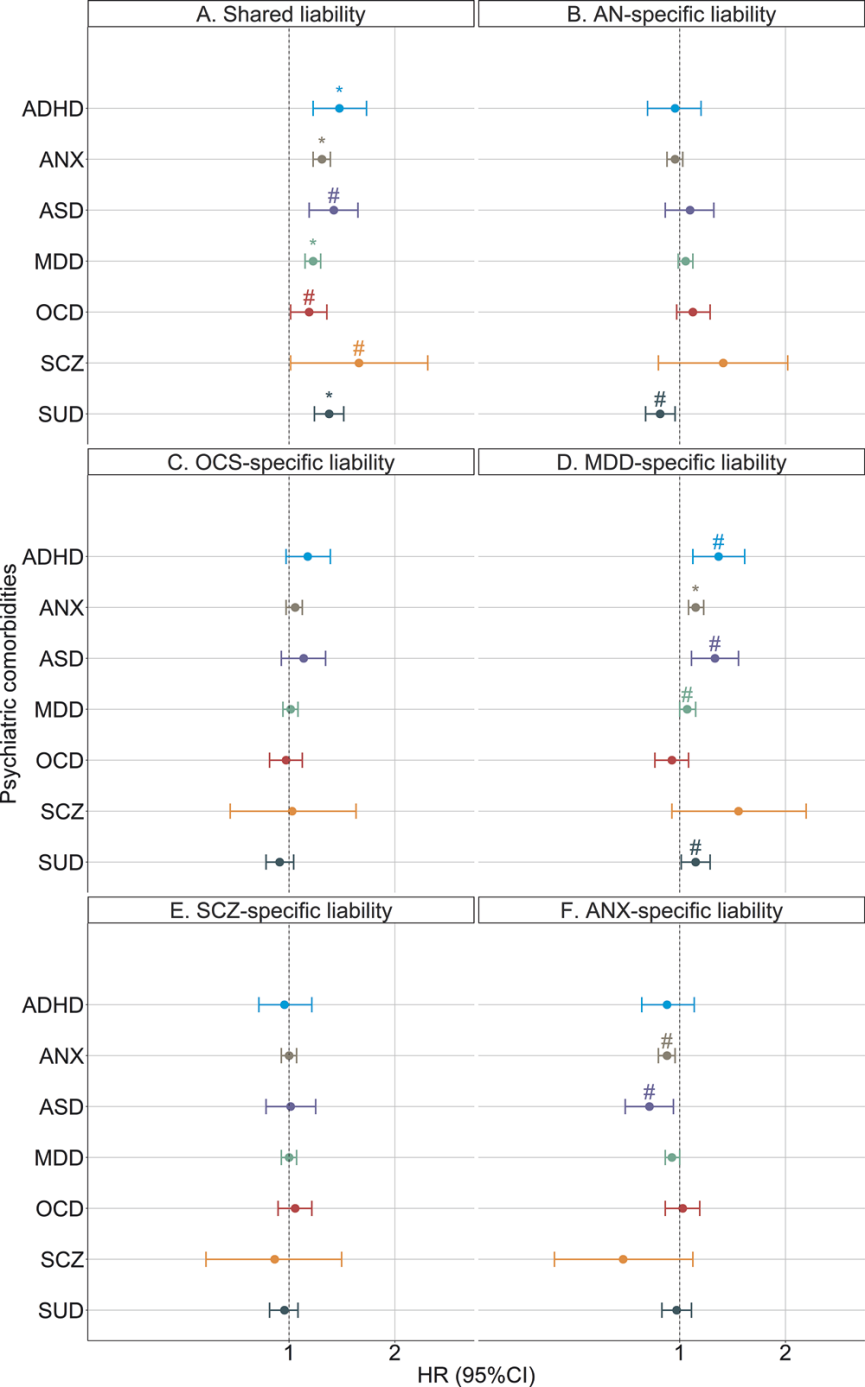
Association between transdiagnostic genetic liabilities and psychiatric comorbidities from the Cox regression models. Results are derived from quasi-Possion regression models based on 4028 AN cases with ADHD, ANX, ASD, MDD, OCD, SCZ and SUD as the outcome variables. For each of the seven outcomes, we constructed six models with PRS_shared_ (**A**), PRS_AN-specific_ (**B**), PRS_OCS-specific_ (**C**), PRS_MDD-specific_ (**D**), PRS_SCZ-specific_ (**E**) and PRS_ANX-specific_ (**F**) as exposure variable, respectively. Incidence rate ratios indicate the risk estimates for +1 SD increase of PRS. Sex, birth year and first 10 ancestry-informative principal components were adjusted for in all models. The points represent hazard ratio estimates, and the error bars indicate 95% confidence intervals. “*” represents association that remained significant after Bonferroni correction. “#” represents trending association at *P* < 0.05 but was not significant after Bonferroni correction. PRS polygenic risk scores, AN anorexia nervosa, OCS obsessive-compulsive symptoms, OCD obsessive-compulsive disorder, SCZ schizophrenia, MDD major depressive disorder, ANX anxiety disorders, ASD autism spectrum disorder, ADHD attention-deficit hyperactivity disorder, SUD substance use disorder.

Increased PRS_shared_ was associated with increased risk of all psychiatric comorbidities at *P* < 0.05, with effect sizes ranging from +14–+58% excess risk (Fig. 3A; Table S9). Increased PRS_MDD-specific_ was associated with elevated risks for ADHD, ANX, ASD, MDD and SUD at *P* < 0.05 (Fig. 3D; Table S9). Most associations did not withstand Bonferroni correction, except for the associations for PRS_shared_ with ADHD, ANX, MDD and SUD as well as the one between PRS_MDD-specific_ and ANX (Fig. 3; Table S9).

### Association between gSEM-derived PRSs and AN symptomatology at first clinical visit

**Fig. 4:**
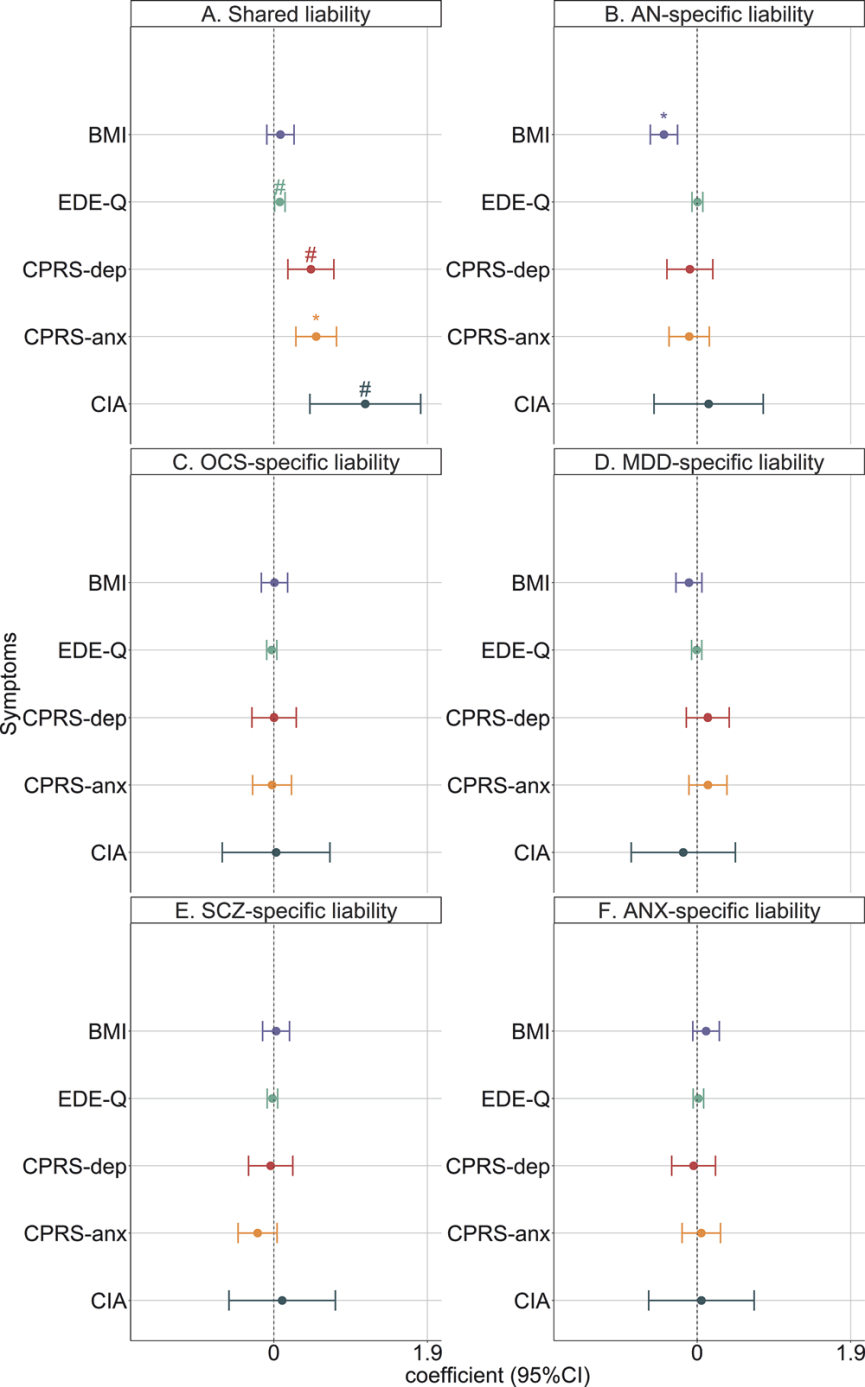
Association between transdiagnostic genetic liabilities and AN symptomatology from the linear regression models. Results are derived from linear regression models with BMI, EDE-Q score, CPRS-depression score, CPRS-anxiety score and CIA score as the outcome variables. The analyses were based on 1934 ANGI-SE AN cases with available data on symptomatology in Stepwise Quality Register. For each of the five outcomes, we constructed six models with PRS_shared_ (**A**), PRS_AN-specific_ (**B**), PRS_OCS-specific_ (**C**), PRS_MDD-specific_ (**D**), PRS_SCZ-specific_ (**E**) and PRS_ANX-specific_ (**F**) as the exposure variable, respectively. Regression coefficients indicate the risk estimates for +1 SD increase of PRS. Sex, birth year and first 10 ancestry-informative principal components were adjusted for in all models. The points represent regression coefficients, and the error bars indicate 95% confidence intervals. “*” represents association that remained significant after Bonferroni correction. “#” represents trending association at *P* < 0.05 but was not significant after Bonferroni correction. PRS polygenic risk scores, AN anorexia nervosa, OCS obsessive-compulsive symptoms, SCZ schizophrenia, MDD major depressive disorder, ANX anxiety disorders, BMI body mass index, CIA Clinical Impairment Assessment, CPRS Comprehensive Psychopathological Rating Scale, CPRS-dep CPRS-depression score, CPRS-anx CPRS-anxiety score, EDE-Q Eating Disorder Examination Questionnaire.

PRS_shared_ was associated with more severe ED symptoms (regression coefficient, 0.07; 95%CI, 0.01–0.14; *P* = 0.03), greater clinical impairment (regression coefficient, 1.13; 95%CI, 0.45–1.82; *P* = 1.25 × 10^−3^), greater self-reported anxiety (regression coefficient, 0.52; 95%CI, 0.27–0.77; *P* = 4.43 × 10^−5^) and depression (regression coefficient, 0.46; 95%CI, 0.17–0.74; P = 1.71 × 10^−3^) at *P* < 0.05 (Fig. 4A; Table S10). Statistically significant associations were also observed between PRS_AN-specific_ and lower BMI (regression coefficient, −0.41; 95%CI, −0.58–−0.24; *P* = 1.78 × 10^−6^) at *P* < 0.05 (Fig. 4B; Table S10). After correction, only the associations for PRS_shared_ with self-reported anxiety symptoms and PRS_AN-specific_ with lower BMI remained significant.

## Discussion

This study is the first to leverage transdiagnostic psychiatric genetic liability to dissect the clinical heterogeneity of AN so far. We found that shared psychiatric genetic liability was a consistent predictor of disease burden, risk of psychiatric comorbidity and clinical impairment within AN, whereas AN-specific liability was not a good indicator of its clinical course. These findings provide a novel perspective on the heterogeneous etiology and clinical course of AN.

We observed that only AN-specific liability and shared liability were associated with an elevated AN risk. However, all other gSEM-derived trait-specific PRSs were associated with either a statistically non-significant or reduced risk of AN. This offers a completely different perspective on AN etiology compared to the consistently positive relationships between unmodified single-disorder PRSs and AN risk. Since the single-disorder PRSs can capture both shared and trait-specific genetic factors, their associations with increased AN risk might be attributed to the shared psychiatric genetic component. These findings suggest that both general psychiatric and AN-specific genetic factors are underlying the onset of AN.

Shared psychiatric liability was associated with greater cumulative disease burden, elevated risks of psychiatric comorbidities and more severe symptoms and clinical impairment in AN. The findings are consistent with previous studies revealing a positive association between family history of SCZ and AN disease burden, as well as between OCD-AN shared liability and AN symptoms [11, 12]. According to the “p factor” theory, there are shared psychopathological mechanisms underlying multiple psychiatric disorders which could be captured by a common latent factor [30]. The positive association between shared liability and psychiatric disease burden in AN offers biological evidence for the “p-factor” in this population. Furthermore, we shed new light on the genetic underpinnings of this “p-factor” by revealing the relationship between shared liability and other clinical outcomes (i.e. more somatic diagnoses, more medication prescriptions, more inpatient days, more severe symptoms and clinical impairment) in AN.

AN-specific liability did not show statistically significant associations with most of the clinical features in AN. It is interesting that AN-specific liability was a predictor for AN status as expected, but not for its clinical outcomes, suggesting that distinct genetic mechanisms may underlie the onset and prognosis of AN. However, its associations with lower BMI and more inpatient days due to EDs suggest a potential role of AN-specific liability as a predictor for AN-specific symptoms and severity, which is consistent with a previous study revealing that AN PRS showed better performance in predicting ED severity compared to a cross-disorder PRS [12]. Given the low heritability of AN-specific genetic effects (Table S11), the analyses should be replicated when larger GWASs emerge.

MDD-specific liability was associated with greater disease burden within AN. Early-life depression has previously been found to be associated with an increased risk of somatic conditions, possibly due to shared inflammatory or metabolic mechanisms [31–34]. Moreover, genetic overlap has been observed between MDD and endocrine disorders, obesity, and inflammatory cytokines [33, 35–38]. The association between MDD-specific genetic risk and poor health status in AN might be mediated by these biological mechanisms. Our findings should be interpreted with caution given the low heritability of disorder-specific genetic effects and still growing sample sizes of source GWASs.

## Limitations

Our study has several limitations. Firstly, the source GWAS for AN has relatively small sample size, and the GWAS for OCS is based on self-reported obsessive compulsive symptoms, so our analyses should be replicated when larger and diagnosis-based GWASs for AN and OCD respectively are available. Secondly, although our gSEM model fit was good and we showed that the gSEM-derived components had acceptable validity in our study population (Supplementary Results, Table S12), the results for ANX-specific liability should be interpreted with caution since the correlation between PRS_ANX-specific_ and PRS_ANX_ was not strong (Supplementary Results, Table S13 and Fig. S2). Moreover, data on AN symptoms were missing for a considerable proportion of participants and were measured by self-report questionnaires which might lead to underestimates due to subjective denial of symptoms [39]. Given the limited follow-up time, our data might not cover first diagnoses of some psychiatric comorbidities with an early onset age, such as ADHD and ASD. Finally, effects from rare variants and structural variations were not considered in the current study.

## Conclusions

Our findings show that shared instead of AN-specific liability was a strong predictor for adverse outcomes of AN, suggesting that genetic risk profiles for AN diagnosis may be distinct from those for AN outcomes. We provide a novel perspective on the heterogenous clinical outcomes within AN by identifying clinically relevant genetic components.

## Supplementary information

Supplementary materials

## Data Availability

GWAS summary statistics for anorexia nervosa, obsessive-compulsive symptoms, schizophrenia, major depressive disorder and anxiety disorders are publicly available at the Psychiatric Genomics Consortium data downloads portal: https://pgc.unc.edu/for-researchers/download-results/. The individual genotype data for ANGI-SE participants are deposited in dbGaP (http://www.ncbi.nlm.nih.gov/gap) under accession number phs001541.v1.p1. However, their linked information from the Swedish National Registers cannot be shared publicly due to legal and ethical restrictions.
