# Supplementary material for: Leveraging transdiagnostic genetic liability to psychiatric disorders to dissect clinical outcomes of anorexia nervosa
Source: Mol Psychiatry. 2025 Sep 23;31(3):1475–84. doi: 10.1038/s41380-025-03264-x (PMC12916308; doi:10.1038/s41380-025-03264-x)

**Supplementary materials**

**Leveraging transdiagnostic genetic liability to psychiatric disorders to dissect clinical outcomes of anorexia nervosa**

**Zheng-An Lu, MS^1^; Alexander Ploner, PhD^1^; Andreas Birgegård, PhD^1^; Eating Disorders Working Group of the Psychiatric Genomics Consortium; Mikael Landén, PhD^1,2^; Cynthia M. Bulik, PhD^1,3,4^; Sarah E. Bergen, PhD^1^**

1 Department of Medical Epidemiology and Biostatistics, Karolinska Institutet, Stockholm, Sweden.

2 Institute of Neuroscience and Physiology, Sahlgrenska Academy at University of Gothenburg, Gothenburg, Sweden.

3 Department of Psychiatry, University of North Carolina at Chapel Hill, Chapel Hill, NC, USA.

4 Department of Nutrition, University of North Carolina at Chapel Hill, Chapel Hill, NC, USA

**Supplementary Methods**

**Supplementary Results**

**Table S1. Basic information on source GWAS studies**

**Table S2. Extracted ICD-10 codes for calculating number of clinical diagnoses and inpatient days**

**Table S3. Extracted ATC codes for calculating number of medication prescriptions Table S4. Extracted ICD codes for psychiatric comorbidities**

**Table S5. Descriptions of the fitted models**

**Table S6. Descriptive characteristics of individuals with anorexia nervosa in ANGI-SE**

**Table S7. Association between source GWAS polygenic risk scores (PRS) and risk of anorexia nervosa (AN) from logistic regression analyses**

**Table S8. Association between transdiagnostic genetic liabilities and number of unique clinical diagnoses, medication prescriptions and inpatient days from quasi-Poisson regression analyses**

**Table S9. Association between gSEM-derived polygenic risk scores and risks of psychiatric comorbidities within anorexia nervosa from Cox regression analyses**

**Table S10. Association between gSEM-derived polygenic risk scores and symptomatology of anorexia nervosa at first clinical visit from linear regression analyses**

**Table S11. Heritability of gSEM-derived genetic components from LDSC Table S12. Model fitting statistics of common-factor gSEM model**

**Table S13. Pearson's correlations between gSEM-derived PRSs and source GWAS PRSs in the ANGI-SE data**

**Fig.S1. GSEM models applied to source GWASs to extract shared and disorder- specific liability components**

**Fig.S2. Pearson's correlations between gSEM-derived PRSs and source GWAS PRSs in the ANGI-SE data**

**Supplementary Methods**

*Recruitment of participants in the Swedish arm of the Anorexia Nervosa Genetics Initiative (ANGI-SE)*

In Sweden, the AN cases were recruited from the Riksät-National Quality Register for Eating Disorders Treatment [ANGI-SE(Riksät)], Stockholm Centre for Eating Disorders [ANGI-SE(SCÄ)], the community [ANGI-SE(Community)] and the LifeGene study (1). Riksät includes information from individuals seeking treatment for an eating disorder in Sweden since 1999 (1). Cases from the community were approached using traditional media, social media and the Swedish ANGI website [(www.angi.se)](http://www.angi.se/) (1). LifeGene (https[://www.lifegene.se),](http://www.lifegene.se/) an ongoing study evaluating the impact of genes and environment on health, also provided AN cases (2). Status of AN was determined based on a DSM-IV-based AN diagnosis or responses to the ED100K-v1 questionnaire (1). Statistics Sweden provided age- and sex-matched controls who were matched to cases in Riksät quality register, and the LifeGene study provided an additional 3, 000 archived controls (1). All participants in the ANGI-SE provided informed consent and a blood DNA sample (1). ANGI-SE was approved by the regional Ethical Review board in Stockholm (DNR 2013/112- 31/2). Details on recruitment procedures were reported previously (1).

*Phenotypes*

Number of clinical diagnoses and inpatient days were derived from the National

Patient Register (NPR) using the ICD-10 (The 10th version of the International Classification of Disease) diagnostic codes shown in Table S2. Clinical diagnoses included all the records from outpatient visits, while inpatient days included all the records from inpatient visits. Inpatient days due to EDs only included hospitalization periods with EDs as the main diagnosis. Drug prescriptions were derived from the National Prescribed Drug Register (NPDR) using the ATC codes presented in Table S3. Data on psychiatric comorbidities were derived from the NPR and Cause of Death Register (CDR) using the ICD-10 diagnostic codes available in Table S4. Symptoms of AN at first clinical visit, including body mass index, Eating Disorders Examination Questionnaire (EDE-Q), depression subscale score from the Comprehensive Psychopathological Rating Scale (CPRS), anxiety subscale score from the CPRS and Clinical Impairment Assessment (CIA) scores, were derived from National Eating Disorder quality register (Stepwise). The information for either of the five symptoms was only available for 1,934 AN cases in the ANGI-SE sample. Demographic variables, including birth year and sex, were extracted from the Total Population Register (TPR).

**Statistical analyses**

*Genomic SEM (gSEM)*

We performed gSEM with the R package “GenomicSEM” (“0.0.5”). Codes and technical details are available online (https://github.com/GenomicSEM/GenomicSEM/).

We prepared the dataset for gSEM analyses in the following four steps: (1) we employed the “munge()” function to combine the formatted GWAS datasets into one munged dataset and convert it to the format accepted by LD-score regression (LDSC); (2) we employed the “ldsc()” function to run multivariable LDSC based on the munged summary statistics to obtain the genetic covariance matrix and sampling covariance matrix; (3) we employed “sumstats()” function to prepare the input dataset for gSEM. In this step, SNPs and SNP effects were aligned across phenotypes to ensure same reference allele in all cases. Moreover, SNP effects and SEs were standardized into scales relative to unit-variance scaled phenotypes. As the GWASs for AN, SCZ and MDD were based on logistic regression on binary outcomes and the standard errors were for logistic beta, we set their parameters as: se.logit = T, OLS = F; linprob = F; as the GWAS for OCS was based on continuous symptom outcome, we set its parameters as: se.logit = F, OLS = T; linprob = F; since the GWAS for ANX was a meta-analysis of continuous and binary outcomes, we set its parameters as: se.logit = F, OLS = T; linprob = F (3). We filtered SNPs with an imputation info score < 0.6 or a minor allele frequency < 1% by

setting “info.filter” = 0.6 and “maf.filter” = 0.1, and excluded SNPs not present in

either source GWAS datasets or HapMap3. A total of 2,284,418 SNPs were included for gSEM model construction.

After the preparation of input dataset, we first constructed a common factor model (Fig.S1a). The model had two parts: (1) a latent variable F1 indexing shared genetic

component was defined based on 5 source GWASs; (2) the F1 variable was regressed on each individual SNP. We extracted the regression coefficient for each SNP and referred to it as the “shared” effect, indicating contribution of the SNP to general liability to the five disorders. The model was estimated using DWLS estimation.

Next, we constructed a common factor and residual variance model for each trait (Fig.S1b-f). Each model had two parts: (1) a latent variable F1 indexing shared genetic component was defined based on five source GWASs; (2) the residual variance of a specific trait after accounting for F1 was regressed on each individual SNP. We extracted the regression coefficient of residual variance of

each disorder on each SNP and referred to it as the “trait-specific” effect, indicating the contribution of the SNP on AN-specific, OCS-specific, MDD-specific, SCZ- specific and ANX-specific genetic liability after controlling for the shared genetic component. The models were estimated using DWLS estimation.

*Target genotype dataset quality control and imputation*

The genotyping procedures of the ANGI-SE dataset were described in detail in previous research (1). The quality control and imputation were performed according to the RICOPILI pipeline (4). SNPs were excluded from analyses when they showed deviations from Hardy-Weinberg equilibrium (P < 1 × 10-6), had a missing rate > 5% or minor allele frequency < 1%. Individuals with genotype missingness rate >

5%, mismatched genetic sex and reported sex, extreme genetic heterogeneity, high familial relatedness (kinshio coefficient> 0.2), and ancestry outliers were excluded from analyses, leaving a total of 7,874 individuals (4,028 cases and 3,846 controls). Following QC, the Haplotype Reference Consortium panel was used as the reference panel for imputation, leaving a total of 7, 357, 567 SNPs in the genotype dataset (4).

*Polygenic risk score (PRS) calculation*

The target dataset was the genotype data from 7,874 individuals in ANGI-SE. The base summary statistic datasets were the shared and trait-specific effects from gSEM. Before polygenic risk score calculation, we first excluded all multiallelic and duplicated SNPs from the base GWAS datasets. After that, we harmonized the genotype target dataset and base datasets (gSEM-derived genetic effects) by aligning the effect alleles, excluding SNPs with mismatched alleles, and only retaining SNPs that were included in both base and target datasets, leaving a total of 2,208,661 SNPs for PRS calculation. We generated PRSs using PRSice software (version 2.3.5) (5). We clumped the SNPs at r^2^ < 0.1 within 250 kb and aggregated their effects at different p-value thresholds (5e-8, 1e-6, 1e-4, 0.001,0.01, 0.05, 0.1, 0.2, 0.5, 1) using the PRS-PCA method (6). The final PRSs utilized in association analyses were the standardized first components derived from principal component analyses (PCA) of PRSs at all thresholds. We performed PCA and standardization

with R functions “princomp()” and “scale()”, respectively.

*Association analyses*

To investigate the association between gSEM-derived PRSs and AN status, we conducted logistic regression analyses with binary AN status as the outcome variable among 4,028 AN cases and 3,846 controls. We constructed six models

with PRSshared, PRSAN-specific, PRSOCS-specific, PRSMDD-specific, PRSSCZ-specific and PRSANX-specific as the independent variable, respectively. Odds ratios (ORs) represented the risk estimates

+1 standard deviation (SD) increase of PRS.

To investigate the impact of gSEM-derived PRSs on the cumulative disease burden of AN cases, we conducted quasi-Poisson regression analyses with number of any unique diagnoses, unique somatic diagnoses, unique psychiatric diagnoses, any unique medication prescriptions, unique antipsychotics prescriptions, unique

antidepressants prescriptions, any inpatient days and inpatient days due to eating disorders recorded from January 1, 1997 to December 31, 2018 as outcome variables. For each of the eight outcomes, we constructed six models with PRSshared, PRSAN-specific, PRSOCS-specific, PRSMDD-specific, PRSSCZ-specific and PRSANX-specific as the independent variable, respectively.

We included a log-time offset term in each model to adjust for differences in follow-up time between individuals. Incidence rate ratios (IRRs) represented the risk estimates +1 SD increase of gSEM-derived PRS.

To investigate the impact of gSEM-derived PRSs on risks of psychiatric comorbidities in AN cases, we conducted Cox regression (survival) analyses with onset of OCD, SCZ, MDD, ANX, ASD, ADHD, and SUD as outcomes and age as the underlying timescale. For each of the seven outcomes, we constructed six models with PRSshared, PRSAN-specific, PRSOCS-specific, PRSMDD-specific, PRSSCZ-specific and PRSANX-specific as the independent variable, respectively. Individuals were followed from January 1, 1997 until onset of disorder, death or December 31, 2018, whichever came first. Hazard ratios (HRs) represented the risk estimates +1 SD increase of gSEM-derived PRS.

To investigate the impact of gSEM-derived PRSs on the symptomatology of AN at first visit in AN cases, we performed linear regression analyses with BMI, EDE-Q scores, CIA scores, CPRS-depression and CPRS-anxiety scores as outcome variables. For each of the five outcomes, we constructed six models with PRSshared, PRSAN-specific, PRSOCS-specific, PRSMDD-specific, PRSSCZ-specific and PRSANX-specific as the independent variable, respectively. Regression coefficients represented change in symptoms +1 SD increase of gSEM-derived PRS.

All association analyses were performed in R version 4.2.3. The significance level in all association analyses above was set at two-sided P < .05. In all regression models, we adjusted for birth year, sex, and the first 10 ancestry-informative

principal components. To correct for multiple comparisons, we also employed a Bonferroni-corrected significance level of two-sided P < .05/6 = 8.33 × 10^-3^ for association tests with AN status and two-sided P < .05/120 = 4.17 × 10^-4^ for association tests with AN clinical outcomes.

**Supplementary Results**

*Heritability of gSEM-derived summary statistics from LDSC*

SNP-based heritability of gSEM-derived summary statistics ranged from 0.002 to

0.184 (Table S11).

*Model fitting statistics of gSEM*

The gSEM model fit was good as indicated by the Comparative Fit Indicator (0.99) and Standardized Root Mean Squared Residual (0.05) (Table S12).

*Correlations between gSEM-derived and source GWAS PRSs*

Among 4,028 individuals with AN, PRSshared showed moderate to strong positive correlations with PRSshared, PRSAN-specific, PRSOCS-specific, PRSMDD-specific, PRSSCZ-specific and PRSANX-specific (Table S13). This indicates that the shared genetic component derived from gSEM at least captures some of the pleiotropic genomic risk underlying the

included psychiatric traits. Moreover, most gSEM-derived trait-specific

PRSs were positively correlated with their corresponding source GWAS PRSs

(r=0.46-0.86), except for ANX (Table S13). In contrast, the cross-trait

correlations between trait-specific PRSs and other source GWAS PRSs were mostly weak and not statistically significant.

**References:**

1. Thornton LM, Munn-Chernoff MA, Baker JH, et al. The Anorexia Nervosa Genetics Initiative (ANGI): Overview and methods. Contemp Clin Trials. Nov 2018;74:61-69. doi:10.1016/j.cct.2018.09.015
2. Almqvist C, Adami HO, Franks PW, et al. LifeGene--a large prospective population-based study of global relevance. Eur J Epidemiol. Jan 2011;26(1):67-

77. doi:10.1007/s10654-010-9521-x

1. https://github.com/GenomicSEM/GenomicSEM/
2. Lam M, Awasthi S, Watson HJ, et al. RICOPILI: Rapid Imputation for COnsortias PIpeLIne. Bioinformatics. Feb 1 2020;36(3):930-933. doi:10.1093/bioinformatics/btz633
3. Choi SW, O'Reilly PF. PRSice-2: Polygenic Risk Score software for biobank-scale data. Gigascience. Jul 1 2019;8(7)doi:10.1093/gigascience/giz082
4. Choi SW, Mak TS, O'Reilly PF. Tutorial: a guide to performing polygenic risk score analyses. Nat Protoc. Sep 2020;15(9):2759-2772. doi:10.1038/s41596- 020-0353-1

**Table S1. Basic information on source GWAS studies**

| **Traits** | **Title** | **Reference** | **No. of cases** | **No. of controls** | **Ancestry** |
| --- | --- | --- | --- | --- | --- |
| Anorexia nervosa | Genome-wide association study | Watson et al, | 12,887 | 51,732 | European |
| (without ANGI-SE | identifies eight risk loci and | Nat Genet, |  |  |  |
| samples, Ncases = 4,105, | implicates metabo-psychiatric | 2019 |  |  |  |
| Ncontrols = 3,793) | origins for anorexia nervosa |  |  |  |  |
| Obsessive-compulsive | Genome-Wide Association | Strom et al, | 33,943 | | European |
| symptoms | Study of Obsessive-Compulsive | Mol |  | |  |
|  | Symptoms including 33,943 | Psychiatry, |  | |  |
|  | individuals from the general Population | 2024 |  | |  |
| Major depressive disorder (without 23andMe) | Depression pathophysiology, risk prediction of recurrence and comorbid psychiatric disorders using genome-wide analyses | Als et al, Nat Med, 2023 | 294,322 | 741,438 | European |
| Schizophrenia (European subset) | Mapping genomic loci implicates genes and synaptic biology in schizophrenia | Trubetskoy et al, Nature, 2022 | 53,386 | 77,258 | European |
| Anxiety disorders (European subset) | Gene discovery and biological insights into anxiety disorders from a large-scale multi- ancestry genome-wide  association study | Friligkou, Nat Genet, 2024 | 87,517 | 1,008,941 | European |

**Table S2. Extracted ICD-10 codes for calculating number of clinical diagnoses and inpatient days**

| **Clinical diagnoses - Any diagnoses** | **Clinical diagnoses**  **- Somatic diagnoses** | **Clinical diagnoses - Psychiatric diagnoses** | **Inpatient days** | **Inpatient days due to eating disorders** |
| --- | --- | --- | --- | --- |
| C0-C9,D0-D9, | C0-C9,D0-D9, E0- | F0-F9 | C0-C9,D0-D9, | F500, F501, F502, |
| E0-E9,F0-F9, | E9, G0-G9, H0- |  | E0-E9,F0-F9, | F503, F509 |
| G0-G9, H0-H9, | H9, I0-I9, J0-J9, |  | G0-G9, H0-H9, |  |
| I0-I9, J0-J9, K0- | K0-K9, L0-L9, |  | I0-I9, J0-J9, |  |
| K9, L0-L9, M0- | M0-M9, N0-N9, |  | K0-K9, L0-L9, |  |
| M9, N0-N9, O0- | O0-O9, A01, A02, |  | M0-M9, N0- |  |
| O9, A01, A02, | A07, A08, A09, |  | N9, O0-O9, |  |
| A07, A08, A09, | A10, A11, A12, |  | A01, A02, A07, |  |
| A10, A11, A12, | A14 |  | A08, A09, A10, |  |
| A14 |  |  | A11, A12, A14 |  |

ICD-10 diagnostic codes were extracted from National Patient Register for calculating the number of clinical diagnoses (any, somatic and psychiatric) and inpatient days (any and due to eating disorders) in Table S8 and Fig. 2. The clinical diagnoses were based on records of outpatient visits. The inpatient days were based on the records of inpatient visits.

**Table S3. Extracted ATC codes for calculating number of medication prescriptions**

| **Any prescriptions** | **Antipsychotics** | **Antidepressants** |
| --- | --- | --- |
| A | N05A | N06A |
| B01, B02, B03 |  |  |
| C01, C02,C03 |  |  |
| G03 |  |  |
| H01,H02,H03,H04 |  |  |
| M05 |  |  |
| N03, N04, N05, N06 |  |  |

ATC codes were extracted from the National Prescribed Drug Register for calculating the number of medication prescriptions in Table S8 and Fig. 2.

**Table S4. Extracted ICD-10 codes for psychiatric comorbidities**

| **Psychiatric comorbidities** | **ICD-10 codes** |
| --- | --- |
| Schizophrenia | F200-F206, F208-F209, F231,F232,F250-F252,F258,F259 |
| Major depressive disorder | F320-F323, F328-F329, F330-F334,F338-F339, F348-F349, F380- F381, F388, F39 |
| Obsessive-compulsive disorder | F420-F422, F428, F429 |
| Anxiety disorders | F400-F402,F408-F409, F410-F413, F418-F419 |
| Autism spectrum disorder | F840, F841, F845 |
| Attention-deficit hyperactivity  disorder | F900-F901, F908-F909 |
| Substance use disorder | F10-F19 |

ICD-10 codes for psychiatric comorbidities were extracted from the National Patient Register (NPR) based on both outpatient and inpatient records, and Cause of Death Register (CDR) in Table S9 and Fig. 3.

**Table S5. Descriptions of the fitted models**

| **Outcome** | **Exposure** | **Regression model** | **Number of outcomes** | **Number of exposure** | **Number of models** | **Formula** | **Table** |
| --- | --- | --- | --- | --- | --- | --- | --- |
| **AN status** | PRSgSEM | Logistic regression | 1 | 6 | 6 × 1 = 6 | Binary AN status  ~ PRSgSEM  + Sex  + Birthyear  + PCs 1–10 | Table 1 |
| **Clinical outcomes of AN** | | | | | | | |
| **Cumulative disease burden:** number of unique diagnoses (general, somatic, and psychiatric), number of unique medication prescriptions (any, antipsychotics, and antidepressants), and number of inpatient days (any and due to eating disorders) | PRSgSEM | Quasi- Poisson regression | 8 | 6 | 6 × 8 = 48 | Number of events ~ PRS_gSEM_ +  Sex  + Birthyear  + PCs 1–10  +  offset(log(fo llow-up time)) | Table S8; Fig. 2 |
| **Psychiatric comorbidities:** OCD, SCZ, MDD, ANX, ASD, ADHD, and SUD | PRSgSEM | Cox regression | 7 | 6 | 6 × 7 = 42 | Surv(start age, end age, psychiatric comorbidity  ) ~ PRSgSEM  + Sex  + Birthyear  + PCs 1–10 | Table S9; Fig. 3 |
| **AN symptoms at first visit:** BMI, EDE-Q scores, CIA scores, CPRS-  depression and CPRS-anxiety | PRSgSEM | Linear regression | 5 | 6 | 6 × 5 = 30 | Symptom ~ PRS_gSEM_ +  Sex  + Birthyear  + PCs 1–10 | Table S10;  Fig. 4 |

*PRS* polygenic risk score, *gSEM* Genomic Structural Equation Model, *PC* principal components, *AN* anorexia nervosa, *OCD* obsessive-compulsive disorder, *SCZ* schizophrenia, *MDD* major depressive disorder, *ANX* anxiety disorders, *ASD* autism spectrum disorder, *ADHD* attention-deficit hyperactivity disorder, *SUD* substance use disorder, *BMI* body mass index, *CIA* Clinical Impairment Assessment, *CPRS* Comprehensive Psychopathological Rating Scale, *EDE-Q* Eating Disorder Examination Questionnaire.

**Table S6. Descriptive characteristics of individuals with anorexia nervosa in ANGI-SE**

| **Characteristics** | **ANGI-SE cases (N = 4,028)** |
| --- | --- |
| **Sex female, N (%)** | 3,947 (98.0) |
| **Birth year, mean (± SD)** | 1985 ± 9 |
| **Disease burden, median number of records (Q1,Q3)** | |
| Unique diagnoses (somatic + psychiatric) | 7 (3, 11) |
| Unique somatic diagnoses | 3 (1, 6) |
| Unique psychiatric diagnoses | 2 (1, 5) |
| Inpatient days | 4 (0, 25) |
| Inpatient days due to EDs | 0 (0, 1) |
| Unique prescriptions | 7 (4, 12) |
| Unique antipsychotic prescriptions | 0 (0, 0) |
| Unique antidepressant prescriptions | 1 (0, 3) |
| **Psychiatric comorbidities, N/100 person years (95% confidence interval)** | |
| Obsessive-compulsive disorder | 0.40 (0.36 - 0.45) |
| Schizophrenia | 0.03 (0.02 - 0.04) |
| Anxiety disorders | 1.96 (1.86 - 2.06) |
| Autism spectrum disorder | 0.20 (0.17 - 0.24) |
| Attention-deficit hyperactivity disorder | 0.17 (0.14 - 0.20) |
| Substance use disorder | 0.60 (0.55 - 0.66) |
| Major depressive disorder | 2.51 (2.39 - 2.63) |
| **Symptomatology at first clinical visit, median score (Q1,Q3)** | |
| BMI (N = 1,873) | 17.7 (16.1, 19.8) |
| EDE-Q - total score (N = 1,932) | 3.8 (2.7, 4.7) |
| CPRS - depression subscale score (N = 1,319) | 11.0 (7.5, 14.5) |
| CPRS - anxiety subscale score (N = 1,319) | 9.5 (7.0, 12.5) |
| CIA score (N = 1,132) | 30.0 (22.0, 37.0) |

Data on symptomatology were available for 1,934 ANGI-SE anorexia nervosa cases in Stepwise quality register. Mean (SD) for birth year = 1988 (8.3). N (%) for females = 1,900 (98.2%). The number of individuals with data for each measure was provided in the brackets. *Q1* first quartile, *Q3* third quartile, *BMI* body mass index, *EDE-Q* Eating Disorder Examination Questionnaire, *CPRS* Comprehensive Psychopathological Rating Scale, *CIA* Clinical Impairment Assessment.

**Table S7. Association between source GWAS polygenic risk scores (PRS) and risk of anorexia nervosa (AN) from logistic regression analyses**

| **PRS** | **Odds ratio (95% confidence interval)** | ***P* value** |
| --- | --- | --- |
| PRSAN | 1.41 (1.34 - 1.49) | 5.56 × 10^-37^***** |
| PRSOCS | 1.08 (1.03 - 1.13) | 2.03 × 10^-3^***** |
| PRSSCZ | 1.19 (1.13 - 1.25) | 2.47 × 10^-10^***** |
| PRSMDD | 1.24 (1.18 - 1.30) | 2.92 × 10^-17^***** |
| PRSANX | 1.29 (1.23 - 1.35) | 2.07× 10^-23^***** |

Results were derived from logistic regression models with status of AN as the outcome variable and PRSAN, PRSOCS, PRSMDD, PRSSCZ and PRSANX as exposure variable, respectively. The analyses were based on 7,874 individuals from ANGI-SE (4,028 cases and 3,846 controls) adjusting for birth year, sex and first 10 ancestry-informative principal components. Odds ratios represented the risk estimates per one standard deviation increase of source GWAS PRS. The Bonferroni-corrected significance level was set at *P* < .05/5

= .01. * represents association that remained significant after Bonferroni correction. *AN* anorexia nervosa, *OCS* obsessive-compulsive symptoms, *SCZ* schizophrenia, *MDD* major depressive disorder, *ANX* anxiety disorders.

**Table S8. Association between transdiagnostic genetic liabilities and number of unique clinical diagnoses, medication prescriptions and inpatient days from quasi-Poisson regression analyses**

| **Polygenic risk scores** | **Outcomes** | **Incidence rate ratio** | **95% Confidence interval** | ***P* value** |
| --- | --- | --- | --- | --- |
| PRSshared | Any diagnoses | 1.12 | 1.09 - 1.15 | 1.82 × 10^-18^* |
|  | Somatic diagnoses | 1.09 | 1.06 - 1.13 | 7.01 × 10^-8^* |
|  | Psychiatric diagnoses | 1.16 | 1.12 - 1.21 | 5.46 × 10^-16^* |
|  | Any prescriptions | 1.12 | 1.09 - 1.15 | 1.80 × 10^-18^* |
|  | Antipsychotics | 1.27 | 1.17 - 1.38 | 8.06 × 10^-9^* |
|  | Antidepressants | 1.15 | 1.11 - 1.20 | 3.78 × 10^-15^* |
|  | Inpatient days | 1.19 | 1.08 - 1.32 | 5.79 × 10^-4^ ^#^ |
|  | Inpatient days due to EDs | 1.12 | 0.99 - 1.27 | 7.73 × 10^-2^ |
| PRSAN-specific | Any diagnoses | 0.99 | 0.96 - 1.02 | .43 |
|  | Somatic diagnoses | 0.98 | 0.95 - 1.01 | .18 |
|  | Psychiatric diagnoses | 1.00 | 0.97 - 1.04 | .86 |
|  | Any prescriptions | 0.99 | 0.96 - 1.01 | .30 |
|  | Antipsychotics | 1.00 | 0.92 - 1.08 | .99 |
|  | Antidepressants | 0.99 | 0.96 - 1.03 | .59 |
|  | Inpatient days | 1.04 | 0.94 - 1.15 | .44 |
|  | Inpatient days due to EDs | 1.17 | 1.04 - 1.32 | 9.75 × 10^-3#^ |
| PRSOCS-specific | Any diagnoses | 1.00 | 0.98 - 1.03 | .76 |
|  | Somatic diagnoses | 1.00 | 0.97 - 1.03 | .99 |
|  | Psychiatric diagnoses | 1.01 | 0.97 - 1.04 | .63 |
|  | Any prescriptions | 1.01 | 0.99 - 1.03 | .36 |
|  | Antipsychotics | 1.03 | 0.96 - 1.11 | .39 |
|  | Antidepressants | 1.02 | 0.98 - 1.05 | .34 |
|  | Inpatient days | 1.06 | 0.97 - 1.16 | .21 |
|  | Inpatient days due to EDs | 1.01 | 0.89 - 1.13 | .91 |
| PRSMDD-specific | Any diagnoses | 1.03 | 1.01 - 1.06 | 7.58 × 10^-3#^ |
|  | Somatic diagnoses | 1.01 | 0.98 - 1.04 | .66 |
|  | Psychiatric diagnoses | 1.07 | 1.03 - 1.11 | 1.87 × 10^-4^* |
|  | Any prescriptions | 1.06 | 1.03 - 1.08 | 6.85 × 10^-6^* |
|  | Antipsychotics | 1.14 | 1.06 - 1.24 | 5.43 × 10^-4^ ^#^ |
|  | Antidepressants | 1.09 | 1.05 - 1.13 | 6.63 × 10^-7^* |
|  | Inpatient days | 1.15 | 1.04 - 1.26 | 5.31 × 10^-3#^ |
|  | Inpatient days due to EDs | 1.07 | 0.95 - 1.21 | .26 |
| PRSscz-specific | Any diagnoses | 0.97 | 0.95 - 1.00 | .03 ^#^ |
|  | Somatic diagnoses | 0.96 | 0.93 - 0.99 | .01 ^#^ |
|  | Psychiatric diagnoses | 0.99 | 0.95 - 1.02 | .42 |
|  | Any prescriptions | 0.96 | 0.94 - 0.99 | 4.11 × 10^-3^ ^#^ |
|  | Antipsychotics | 0.99 | 0.91 - 1.07 | .76 |
|  | Antidepressants | 0.97 | 0.94 - 1.01 | .15 |
|  | Inpatient days | 0.94 | 0.85 - 1.04 | .26 |
|  | Inpatient days due to EDs | 0.95 | 0.84 - 1.07 | .40 |

Results are derived from quasi-Possion regression models based on 4,028 ANGI-SE cases with number of

unique clinical diagnoses (any, psychiatric, somatic), prescriptions (any, antipsychotics, antidepressants) and inpatient days (any, due to EDs) as outcomes. For each outcome, we constructed six models with PRSshared, PRSAN-specific , PRSOCS-specific , PRSMDD-specific , PRSSCZ-specific and PRSANX-specific as exposure variable, respectively. Incidence rate ratios indicate the risk estimates for +1 standard deviation increase of PRS. Sex, birth year and first 10 ancestry-informative principal components were adjusted for in all models. The Bonferroni-corrected significance level was set at *P* < .05/120 = 4.17 × 10^-4^. “*” represents association that remained significant after Bonferroni correction. “#” represents trending association at *P* < .05 but was not significant after Bonferroni correction. *PRS* polygenic risk score, *EDs* eating disorders, *IRR* incidence rate ratio, *95%CI* 95% confidence interval, *AN* anorexia nervosa, *SCZ* schizophrenia, *OCS* obsessive- compulsive symptoms, *MDD* major depressive disorder, *ANX* anxiety disorders.

**Table S9. Association between gSEM-derived polygenic risk scores and risks of psychiatric comorbidities within anorexia nervosa from Cox regression analyses**

| **PRS** | **Psychiatric comorbidities** | **Hazard ratio** | **95%**  **confidence interval** | ***P* value** |
| --- | --- | --- | --- | --- |
| PRSshared | ADHD | 1.39 | 1.17 - 1.66 | 2.68 × 10^-4^* |
|  | ANX | 1.24 | 1.17 - 1.31 | 1.02 × 10^-13^* |
|  | ASD | 1.34 | 1.14 - 1.57 | 4.27 × 10^-4^ ^#^ |
|  | MDD | 1.17 | 1.11 - 1.23 | 7.70 × 10^-10^* |
|  | OCD | 1.14 | 1.01 - 1.28 | .03 ^#^ |
|  | SCZ | 1.58 | 1.01 - 2.48 | .05 ^#^ |
|  | SUD | 1.30 | 1.18 - 1.43 | 2.13 × 10^-7^* |
| PRSAN-specific | ADHD | 0.97 | 0.81 - 1.15 | .69 |
|  | ANX | 0.97 | 0.92 - 1.02 | .27 |
|  | ASD | 1.07 | 0.91 - 1.25 | .42 |
|  | MDD | 1.04 | 0.99 - 1.09 | .17 |
|  | OCD | 1.09 | 0.98 - 1.22 | .13 |
|  | SCZ | 1.33 | 0.87 - 2.03 | .18 |
|  | SUD | 0.88 | 0.80 - 0.97 | 7.99 × 10^-3^ ^#^ |
| PRSOCS-specific | ADHD | 1.13 | 0.98 - 1.31 | .09 |
|  | ANX | 1.04 | 0.98 - 1.09 | .18 |
|  | ASD | 1.10 | 0.95 - 1.27 | .20 |
|  | MDD | 1.01 | 0.96 - 1.06 | .66 |
|  | OCD | 0.98 | 0.88 - 1.09 | .73 |
|  | SCZ | 1.02 | 0.68 - 1.55 | .92 |
|  | SUD | 0.94 | 0.86 - 1.03 | .18 |
| PRSMDD-specific | ADHD | 1.29 | 1.09 - 1.53 | 3.23 × 10^-3^ ^#^ |
|  | ANX | 1.11 | 1.06 - 1.17 | 7.98 × 10^-5^* |
|  | ASD | 1.26 | 1.08 - 1.47 | 3.14 × 10^-3^ ^#^ |
|  | MDD | 1.05 | 1.00 - 1.11 | .03 ^#^ |
|  | OCD | 0.95 | 0.85 - 1.06 | .39 |
|  | SCZ | 1.47 | 0.95 - 2.29 | .08 |
|  | SUD | 1.11 | 1.01 - 1.22 | .02 ^#^ |
| PRSSCZ-specific | ADHD | 0.97 | 0.82 - 1.16 | .75 |
|  | ANX | 1.00 | 0.95 - 1.05 | .96 |
|  | ASD | 1.01 | 0.86 - 1.19 | .87 |
|  | MDD | 1.00 | 0.95 - 1.05 | .96 |
|  | OCD | 1.04 | 0.93 - 1.16 | .54 |
|  | SCZ | 0.91 | 0.58 - 1.41 | .67 |
|  | SUD | 0.97 | 0.88 - 1.06 | .48 |
| PRSANX-specific | ADHD | 0.92 | 0.78 - 1.10 | .36 |
|  | ANX | 0.92 | 0.87 - 0.97 | 3.77 × 10^-3^ ^#^ |

|  | ASD | 0.82 | 0.70 - 0.96 | .01 ^#^ |
| --- | --- | --- | --- | --- |
|  | MDD | 0.95 | 0.91 - 1.00 | .05 |
|  | OCD | 1.02 | 0.91 - 1.14 | .72 |
|  | SCZ | 0.69 | 0.44 - 1.09 | .11 |
|  | SUD | 0.98 | 0.89 - 1.08 | .74 |

Results were derived from Cox regression models with ADHD, ANX, ASD, MDD, OCD, SCZ and SUD as the outcome variables. For each outcome, we constructed six models with PRSshared, PRSAN-specific , PRSOCS-specific , PRSMDD-specific , PRSSCZ-specific and PRSANX-specific as the exposure variable, respectively. The analyses were based on 4,028 ANGI-SE cases after adjusting for birth year, sex, and first 10 ancestry- informative principal components. Hazard ratios represent the risk estimates per one standard deviation increase of gSEM-derived PRS. The Bonferroni-corrected significance level was set at *P* < .05/120 = 4.17

× 10^-4^. “*” represents association that remained significant after Bonferroni correction. “#” represents trending association at *P* < .05 but was not significant after Bonferroni correction. *gSEM* Genomic Structural Equation Model, *PRS* polygenic risk scores, *AN* anorexia nervosa, *OCS* obsessive-compulsive symptoms, *OCD* obsessive-compulsive disorder, *SCZ* schizophrenia, *MDD* major depressive disorder, *ANX* anxiety disorders, *ASD* autism spectrum disorder, *ADHD* attention-deficit hyperactivity disorder, *SUD* substance use disorder.

**Table S10. Association between gSEM-derived polygenic risk scores and symptomatology of anorexia nervosa at first clinical visit from linear regression analyses**

| **PRS** | **Symptomatology of AN** | **Regression coefficients (95% confidence interval)** | ***P* value** |
| --- | --- | --- | --- |
| PRSshared | BMI | 0.08 (-0.09 - 0.25) | .35 |
|  | EDE-Q score | 0.07 (0.01 - 0.14) | .03 ^#^ |
|  | CPRS-depression | 0.46 (0.17 - 0.74) | 1.71 × 10^-3^ ^#^ |
|  | CPRS-anxiety | 0.52 (0.27 - 0.77) | 4.43 × 10^-5^ * |
|  | CIA score | 1.13 (0.45 - 1.82) | 1.25 × 10^-3^ ^#^ |
| PRSAN-specific | BMI | -0.41 (-0.58 - -0.24) | 1.78 × 10^-6^ * |
|  | EDE-Q score | 0.00 (-0.06 - 0.07) | .94 |
|  | CPRS-depression | -0.09 (-0.38 - 0.19) | .53 |
|  | CPRS-anxiety | -0.10 (-0.35 - 0.15) | .43 |
|  | CIA score | 0.14 (-0.54 - 0.82) | .68 |
| PRSOCS-specific | BMI | 0.01 (-0.16 - 0.17) | .94 |
|  | EDE-Q score | -0.03 (-0.09 - 0.03) | .38 |
|  | CPRS-depression | 0.00 (-0.27 - 0.28) | .99 |
|  | CPRS-anxiety | -0.02 (-0.27 - 0.22) | .85 |
|  | CIA score | 0.03 (-0.64 - 0.69) | .94 |
| PRSMDD-specific | BMI | -0.10 (-0.26 - 0.06) | .21 |
|  | EDE-Q score | -0.01 (-0.07 - 0.06) | .82 |
|  | CPRS-depression | 0.13 (-0.14 - 0.40) | .34 |
|  | CPRS-anxiety | 0.13 (-0.10 - 0.37) | .27 |
|  | CIA score | -0.17 (-0.82 - 0.47) | .60 |
| PRSSCZ-specific | BMI | 0.03 (-0.14 - 0.19) | .76 |
|  | EDE-Q score | -0.02 (-0.08 - 0.05) | .57 |
|  | CPRS-depression | -0.04 (-0.32 - 0.23) | .77 |
|  | CPRS-anxiety | -0.20 (-0.44 - 0.04) | .10 |
|  | CIA score | 0.10 (-0.56 - 0.76) | .76 |
| PRSANX-specific | BMI | 0.11 (-0.06 - 0.27) | .20 |
|  | EDE-Q score | 0.01 (-0.05 - 0.08) | .67 |
|  | CPRS-depression | -0.04 (-0.32 - 0.23) | .74 |
|  | CPRS-anxiety | 0.05 (-0.19 - 0.29) | .68 |
|  | CIA score | 0.05 (-0.60 - 0.70) | .88 |

Results were derived from linear regression models with BMI, EDE-Q score, CPRS-depression score, CPRS-anxiety score and CIA score as the outcome variables. For each outcome, we constructed six models with PRSshared, PRSAN-specific , PRSOCS-specific , PRSMDD-specific , PRSSCZ-specific and PRSANX-specific as exposure variable, respectively. The analyses were based on 1,934 ANGI-SE AN cases with available data on symptomatology in Stepwise Quality Register. Birth year, sex and first 10 ancestry-informative principal components were adjusted for in all models. Regression coefficients represented the changes in symptom per one standard deviation increase of gSEM-derived PRS. The Bonferroni-corrected significance level was set at *P* < .05/120 = 4.17 × 10^-4^. “*” represents association that remained significant after Bonferroni correction. “#” represents trending association at *P* < .05 but was not significant after Bonferroni correction. *gSEM* Genomic Structural Equation Model, *PRS* polygenic risk scores, *AN* anorexia nervosa, *OCS* obsessive-compulsive symptoms, *SCZ s*chizophrenia, *MDD* major depressive disorder, *ANX* anxiety disorders, *BMI* body mass index, *CIA* Clinical Impairment Assessment, *CPRS* Comprehensive Psychopathological Rating Scale, *EDE-Q* Eating Disorder Examination Questionnaire.

**Table S11. Heritability of gSEM-derived genetic components from LDSC**

| **Genetic components** | **SNP-based heritability (Standard error)** |
| --- | --- |
| Shared | 0.184 (0.006) |
| AN-specific | 0.032 (0.003) |
| OCS-specific | 0.002 (0.002) |
| SCZ-specific | 0.101 (0.005) |
| MDD-specific | 0.040 (0.003) |
| ANX-specific | 0.031 (0.003) |

*AN* anorexia nervosa, *OCS* obsessive-compulsive symptoms, *SCZ* schizophrenia, *MDD* major depressive disorder, *ANX* anxiety disorders.

**Table S12. Model fitting statistics of common-factor gSEM model**

| **Chi-square** | **Df** | ***P* for chi-square** | **Akaike Information Criterion** | **Comparative Fit Index** | **Standardized Root Mean Squared Residual** |
| --- | --- | --- | --- | --- | --- |
| 20.88114 | 5 | 0.000853 | 40.88114 | 0.9935958 | 0.05013695 |

The fitting statistics of a common-factor model with a latent variable F1 indexing shared genetic component defined based on 5 source GWASs.

**Table S13. Pearson's correlations between gSEM-derived PRSs and source GWAS PRSs in the ANGI-SE data**

|  | **Shared** | **AN-**  **Specific** | **SCZ-**  **Specific** | **OCS-**  **specific** | **MDD-**  **specific** | **ANX-**  **Specific** | **AN** | **SCZ** | **OCS** | **MDD** | **ANX** |
| --- | --- | --- | --- | --- | --- | --- | --- | --- | --- | --- | --- |
| **Shared** | 1.00 | 0.09 | 0.25 | -0.02 | 0.14 | -0.37 | 0.25 | 0.54 | 0.09 | 0.71 | 0.76 |
| **AN-**  **specific** | 3.37 ×  10-9 | 1.00 | 0.04 | 0.00 | -0.13 | -0.11 | 0.86 | 0.10 | 0.02 | 0.01 | 0.03 |
| **SCZ-** | 0 | 4.63 × | 1.00 | 0.00 | -0.31 | -0.45 | 0.11 | 0.81 | 0.03 | 0.04 | 0.04 |
| **specific** |  | -3  10 |  |  |  |  |  |  |  |  |  |
| **OCS-**  **specific** | .30 | .80 | .94 | 1.00 | -0.06 | -0.07 | 0.00 | 0.00 | 0.77 | -0.04 | -0.05 |
| **MDD-** | 0 | 0 | 0 | 5.03 × | 1.00 | -0.46 | -0.11 | -0.19 | -0.04 | 0.46 | -0.08 |
| **specific** |  |  |  | -5  10 |  |  |  |  |  |  |  |
| **ANX-** |  | 1.95 × |  | 1.14 × |  |  |  |  |  |  |  |
| **specific** | 0 | -11  10 | 0 | -5  10 | 0 | 1.00 | -0.18 | -0.50 | -0.10 | -0.45 | 0.03 |
| **AN** | 0 | 0 | 2.34 × | .80 | 1.04 × | 0 | 1.00 | 0.23 | 0.05 | 0.14 | 0.16 |
|  |  |  | -12  10 |  | 10-11 |  |  |  |  |  |  |
| **SCZ** | 0 | 8.41× | 0 | .84 | 0 | 0 | 0 | 1.00 | 0.07 | 0.32 | 0.31 |
|  |  | -10 |  |  |  |  |  |  |  |  |  |
|  |  | 10 |  |  |  |  |  |  |  |  |  |
| **OCS** | 1.93 × | .30 | 7.74 × | 0 | 1.40 × | 1.20 × | 1.50× | 7.32 × | 1.00 | 0.06 | 0.05 |
|  | -9  10 |  | -2  10 |  | 10-2 | -10  10 | -3  10 | -6  10 |  |  |  |
| **MDD** | 0 | .55 | 4.31 × | 1.27 × | 0 | 0 | 0 | 0 | 1.45 × | 1.00 | 0.51 |
|  |  |  | -3 | -2 |  |  |  |  | -4 |  |  |
|  |  |  | 10 | 10 |  |  |  |  | 10 |  |  |
| **ANX** | 0 | .03 | 1.99 × | 2.44 × | 1.78 × | 2.77 × | 0 | 0 | 4.81 × | 0 | 1.00 |
|  |  |  | -2  10 | -3  10 | 10-7 | -2  10 |  |  | -4  10 |  |  |

r values are presented in the upper diagonal, and correlation p-values are provided in the bottom diagonal. *AN* anorexia nervosa, *OCS* obsessive-compulsive symptoms, *SCZ* schizophrenia, *MDD* major depressive disorder, *ANX* anxiety disorders.

## Fig.S1: GSEM models applied to source GWASs to extract shared and trait-specific liability components

AN, OCS, MDD, SCZ, and ANX represent anorexia nervosa, obsessive- compulsive symptoms, major depressive disorder, schizophrenia and anxiety disorders source GWAS. “Shared” indicates the genetic liability shared across the five disorders. “AN-diff”, “OCS-diff”, “MDD-diff”, “SCZ-diff”, and “ANX- diff” represent the residual liability after accounting for the shared liability. Red arrow indicates the weight derived from each model. *AN* anorexia nervosa, *OCS* obsessive-compulsive symptoms, *MDD* major depressive disorder, *SCZ* schizophrenia, *ANX* anxiety disorders.

F1 =~ AN + SCZ + OCS + MDD + ANX

# A

F1 ~ SNP

SNP

**Shared**

AN-diff

AN

OCS-

diff

OCS

SCZ-diff

SCZ

ANX-diff

ANX

MDD-

diff

MDD

F1 =~ AN + SCZ + OCS + MDD + ANX

# B

F1 + AN ~ SNP


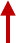


**Shared**

SNP

AN-diff

AN

OCS-

diff

OCS

SCZ-diff

SCZ

ANX-diff

ANX

MDD-

diff

MDD

# C

D

F1 =~ AN + SCZ + OCS + MDD + ANX F1 + OCS ~ SNP

F1 =~ AN + SCZ + OCS + MDD + ANX F1 + MDD ~ SNP


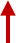


**Shared**

SNP

AN-diff

AN

OCS-

diff

OCS

SCZ-diff

SCZ

ANX-diff

ANX

MDD-

diff

MDD


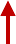


**Shared**

SNP

AN-diff

AN

OCS-

diff

OCS

SCZ-diff

SCZ

ANX-diff

ANX

MDD-

diff

MDD

# E

F

F1 =~ AN + SCZ + OCS + MDD + ANX F1 + SCZ ~ SNP

F1 =~ AN + SCZ + OCS + MDD + ANX


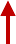


**Shared**

SNP

AN-diff

AN

OCS-

diff

OCS

SCZ-diff

SCZ

ANX-diff

ANX

MDD-

diff

MDD

F1 + ANX ~ SNP


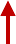


**Shared**

SNP

AN-diff

AN

OCS-

diff

OCS

SCZ-diff

SCZ

ANX-diff

ANX

MDD-

diff

MDD

## Fig.S2: Pearson's correlations between gSEM-derived PRSs and source GWAS PRSs in the ANGI-SE data

Results are derived from Pearson’s correlations between gSEM-derived PRSs and source GWAS PRSs in the ANGI-SE data. Color of the circle indicates the correlation r value. Blue color indicates positive correlation. Red color indicates negative correlation. Size of the circle indicates the correlation p value. Larger circle indicates greater statistical significance. *gSEM* Genomic structrual equation model, *PRS* polygenic risk score, *an* anorexia nervosa, *ocs* obsessive- compulsive symptoms, *mdd* major depressive disorder, *scz* schizophrenia, *anx* anxiety disorders.


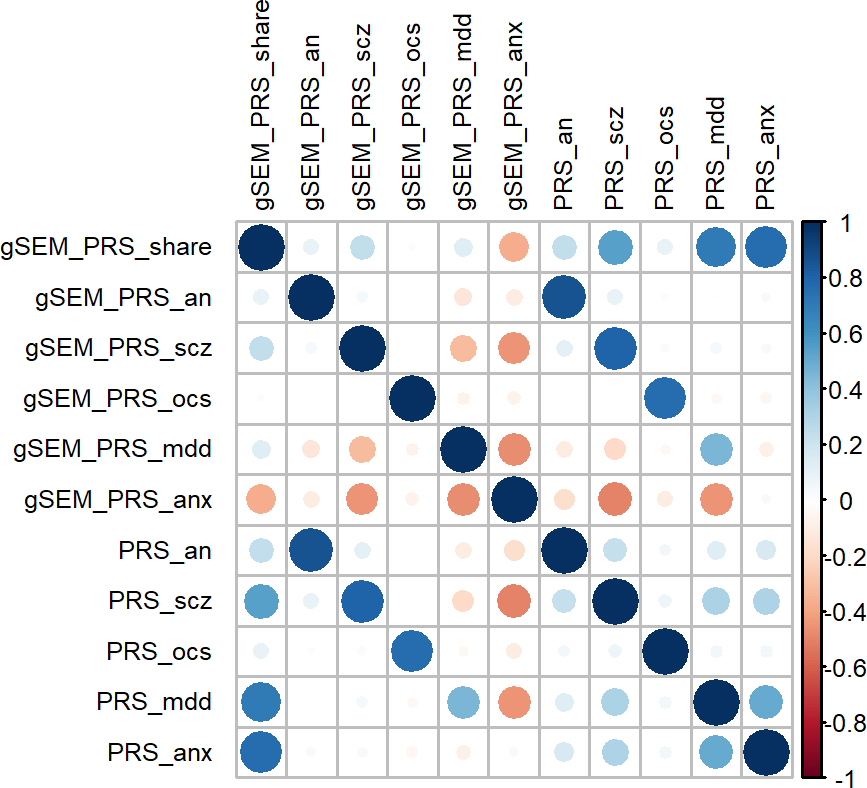

Supplement: Supplementary file 1 — Supplementary materials [file 41380_2025_3264_MOESM1_ESM.docx]
